# Who Reduces Silver? A Critical Review of the Biomolecular Drivers of Fungal-Mediated Silver Nanoparticle Biosynthesis

**DOI:** 10.3390/ijms27136029

**Published:** 2026-07-05

**Authors:** Mislav Vorkapić, Nikolina Filipović, Anamarija Stanković, Ana Amić

**Affiliations:** Department of Chemistry, University of Osijek, Ulica Cara Hadrijana 8/A, 31 000 Osijek, Croatia; mvorkapic@kemija.unios.hr (M.V.); nfilipovic@kemija.unios.hr (N.F.)

**Keywords:** silver nanoparticles, fungal-mediated biosynthesis, fungal secretome, biomolecular mechanisms, nitrate reductase, extracellular synthesis, nanoparticle capping, green nanotechnology

## Abstract

Silver nanoparticles (AgNPs) synthesized via fungal-mediated biosynthesis have gained attention as eco-friendly alternatives to chemically produced nanomaterials, with broad biomedical potential. Fungi represent particularly attractive systems because their secretomes contain diverse biomolecules, including enzymes, proteins, polysaccharides, and secondary metabolites, capable of reducing silver ions and stabilizing the resulting nanoparticles. Despite extensive investigation, the molecular mechanisms underlying fungal-mediated AgNP formation remain poorly defined. This review critically examines the key biomolecular drivers involved in this process, with emphasis on nitrate reductases, oxidoreductases, extracellular proteins, polysaccharides, and secondary metabolites as potential reducing and capping agents. Proposed mechanisms, including nitrate reductase-dependent, superoxide-mediated, and metabolite-driven pathways, are evaluated. The influence of process parameters such as silver nitrate concentration, incubation time, culture medium composition, pH, temperature, and fungal species on nanoparticle yield, size, and stability is also assessed. Analysis of the current literature highlights significant knowledge gaps, including limited application of proteomic and metabolomic approaches, a lack of causal mechanistic studies, and insufficient standardization of experimental protocols. Overall, evidence indicates that fungal AgNP biosynthesis is governed by complex interactions among multiple biomolecular classes rather than a single universal mechanism, underscoring priorities for improving reproducibility, scalability, and mechanistic understanding.

## 1. Introduction

Green synthesis is an environmentally friendly, cost-effective, and sustainable method for the production of nanoparticles. Unlike conventional chemical approaches, biosynthesis utilizes cellular proteins, enzymes, and extracts as reducing agents [1]. This approach minimizes the formation of toxic and harmful byproducts typically associated with chemical synthesis [2]. The green synthesis of silver nanoparticles (AgNPs) specifically refers to the use of biological systems to reduce silver(I) ions (Ag^+^) to elemental silver.

The mechanism of silver ion reduction depends on the synthesis pathway employed. The two most common biosynthetic approaches are intracellular and extracellular synthesis [3]. In intracellular synthesis, Ag^+^ ions are transported into microbial cells, where they are reduced by intracellular enzymes (such as NADH-dependent reductases, nitrate reductases, and other cofactors) and metabolites within the cytoplasm or at the cell membrane. The resulting nanoparticles form and accumulate inside the cell. This approach may provide improved control over nanoparticle size and shape distributions [4], as the confined cellular environment and enzyme specificity regulate nucleation and growth. However, downstream processing is more complex, as nanoparticle recovery requires cell lysis (e.g., ultrasonication, repeated washing, and centrifugation) followed by purification to separate nanoparticles from cellular debris. Consequently, product recovery becomes more labor-intensive and costly [4,5].

In contrast, extracellular synthesis occurs outside the cell, where secreted biomolecules mediate silver ion reduction. Microorganisms release enzymes, proteins, and other reducing agents—including NADH-dependent nitrate reductases, quinones, and phenolic compounds—into the surrounding medium. These biomolecules act as effective reducing agents for Ag^+^ ions [6]. In addition, extracellular proteins with specific molecular weights (e.g., 32 kDa and 35 kDa in *Aspergillus flavus*) have been identified as key contributors to Ag^+^ reduction [7].

Despite extensive research, the molecular drivers underlying fungal-mediated AgNP biosynthesis remain insufficiently understood, warranting a critical evaluation of the biomolecules and mechanisms involved. Silver nanoparticles (AgNPs) have attracted sustained attention because they combine tunable nanoscale properties with broad utility in catalysis, biosensing, bioimaging, and antimicrobial applications. In heterogeneous catalysis, their appeal lies in simple synthetic protocols, often aligned with green chemistry principles, and in their ability to be immobilized on a wide range of supports, including carbon azides, mesoporous Ag/mCTFs, reduced graphene oxides, NHC polymers, carbon nanofibers, and knitting aryl network polymers. Supported AgNPs have been reported in reduction, epoxidation, cyclization of alkynes, N-alkylation, CO_2_ incorporation, photochemical reactions, Diels–Alder cycloaddition, and Suzuki–Miyaura cross-coupling [8], showing that these nanomaterials can contribute to the synthesis of compounds of physiological and therapeutic relevance. Their usefulness extends well beyond catalysis. In biosensing, Ag-based nanostructures can produce rapid and selective optical responses, as illustrated by the colorimetric detection of L-DOPA in biological fluids and by Ag-cluster fluorescent beacons for DNA/RNA detection that turn on upon hybridization to target sequences [9]. These examples show why AgNPs and related Ag clusters are attractive analytical platforms: they can convert molecular recognition into a visible or fluorescent signal while operating in biologically relevant media. In bioimaging, the same fluorescence-activation behavior of silver clusters is especially valuable because it supports light-up probes for nucleic acid targets, offering a route to image specific biological events with high sensitivity [10]. AgNPs are also widely studied for antibacterial applications. Immobilized or entrapped AgNPs are presented as promising antimicrobial materials in which bactericidal action may involve contact killing, ion release, or both. Surface-immobilized AgNPs showed strong disinfection potential with minimal leaching and good reusability, while smaller particles, especially those below 10 nm, displayed enhanced bacteriostatic and bactericidal activity [11,12]. This size dependence is important because it links nanoscale structure directly to antimicrobial performance, helping explain why AgNPs remain a major focus in antibacterial materials research.

## 2. Microbial Platforms for Silver Nanoparticle Biosynthesis

### 2.1. Bacterial and Fungal Systems

Silver ion reduction can be achieved by a variety of microorganisms, among which bacteria and fungi are the most commonly employed [13]. Bacteria are frequently used in AgNP biosynthesis due to their ability to produce nanoparticles with well-defined size, shape, and morphology. In comparison to other biological systems, bacteria offer rapid growth rates and are highly amenable to genetic manipulation [14,15].

**Figure 1:**
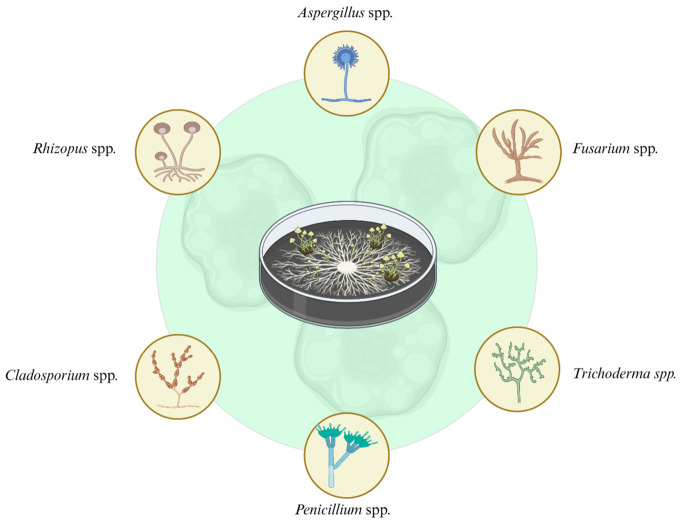
Most commonly used fungi species for extracellular biosynthesis of AgNPs. Created in BioRender. Vorkapić, M. (2026) https://BioRender.com/b7g39iw (accessed on 8 June 2026).

In both bacterial and fungal systems, primary and secondary metabolites play important roles in silver reduction processes [16]. However, fungal-mediated biosynthesis is particularly attractive due to the remarkable diversity of fungal species (Figure 1) and their ability to produce large quantities of AgNPs with controlled morphology and size [17]. This higher yield is largely attributed to the substantial production of extracellular proteins and the high volume of secreted biomolecules present in fungal extracts [18,19].

### 2.2. Advantages of Fungi for AgNP Biosynthesis

Several studies have highlighted the advantages of fungi over bacteria and plants for AgNP biosynthesis. Li et al. (2011) reported that fungi secrete higher quantities of bioactive compounds than bacteria, and that the extracellular nature of fungal synthesis significantly simplifies downstream processing [4]. This observation is supported by Ali et al. (2025), who emphasized that fungi exhibit higher biomass yields and produce abundant extracellular metabolites, thereby enabling more efficient nanoparticle production [2,20]. The scalability of fungal systems is another frequently cited advantage. For example, *Trichoderma reesei* is known for producing large amounts of extracellular enzymes and metabolites substantially higher than many other fungal species, making it a strong candidate for industrial-scale applications. Gemishev et al. (2019) further reinforced this point, noting that fungal systems allow for straightforward large-scale cultivation and simplified biomass harvesting compared to other biological platforms [21].

## 3. Biological Basis of Fungal-Mediated Silver Reduction

Silver nanoparticles are most commonly produced by the chemical reduction of AgNO_3_, in which a reducing agent (most frequently sodium borohydride (NaBH_4_), trisodium citrate (C_6_H_5_Na_3_O_7_), or ascorbic acid (C_6_H_8_O_6_)) converts Ag^+^ to Ag^0^ in the presence of a stabilizing capping agent. NaBH_4_ is the most potent of these reductants and yields small, near-monodisperse particles, though it requires an exogenous stabilizer. Citrate acts simultaneously as a weak reductant, complexing ligand, and electrostatic capping agent and ascorbic acid occupies an intermediate position, producing spherical particles at room temperature with moderate polydispersity [22,23]. Other established routes include the Tollens’ method (reduction of the silver–ammonia complex by an aldehyde), polyol synthesis, and solvothermal approaches [24]. While these methods offer reproducibility and size control, they share fundamental drawbacks: dependence on toxic or environmentally hazardous reagents, generation of chemical waste, and residual surface contamination that compromises biocompatibility [25]. These limitations have motivated the development of green synthesis strategies, in which living organisms replace synthetic reductants.

Fungi represent highly efficient biological systems for the reduction of Ag^+^ into silver nanoparticles (AgNPs), owing to their metabolically versatile and chemically rich secretome. The process of fungal-mediated silver reduction is governed by a complex interplay of biomolecules that function as reducing, stabilizing, and capping agents, enabling both nanoparticle formation and long-term stability.

These biomolecules include enzymes, structural and functional proteins, polysaccharides, and a wide range of secondary metabolites, each contributing through distinct yet often overlapping mechanisms. At the core of this process is the ability of fungal systems to facilitate electron transfer from intracellular or extracellular donors to Ag^+^ ions, leading to their reduction to elemental silver (Ag^0^). Enzymatic systems, particularly oxidoreductases such as nitrate reductase, have been widely recognized as primary drivers of this reduction process [19,21,26,27,28]. However, non-enzymatic biomolecules, including phenolic compounds, sugars, and other metabolites, also significantly contribute to redox reactions through their functional groups and electron-donating capacity [28,29,30,31,32,33]. Despite these insights, the relative contributions and interactions of enzymatic and non-enzymatic systems remain insufficiently resolved at the mechanistic level.

In addition to reduction, stabilization of the formed nanoparticles represents a critical aspect of fungal biosynthesis. Proteins, polysaccharides, and glycoprotein complexes form capping layers on nanoparticle surfaces, preventing aggregation and modulating physicochemical properties such as size, shape, and surface charge [34,35]. Furthermore, the fungal cell wall and extracellular matrix provide a structurally and chemically active microenvironment that supports both nucleation and subsequent nanoparticle growth.

Importantly, the relative contribution of individual biomolecular classes varies depending on fungal species, growth conditions, and environmental parameters, highlighting the species-specific and condition-dependent nature of this process [27,28]. Understanding these biological mechanisms is essential for controlling nanoparticle synthesis and tailoring their properties for targeted applications.

## 4. Biomolecular Drivers of Fungal AgNP Biosynthesis

Fungal-mediated biosynthesis of silver nanoparticles (AgNPs) is governed by a complex and dynamic network of biomolecules present within the fungal secretome. These include enzymes, structural and functional proteins, polysaccharides, and a diverse array of secondary metabolites, all of which contribute, either individually or synergistically, to silver ion reduction, nanoparticle nucleation, growth, and stabilization.

At the core of this process is the ability of fungal systems to mediate redox reactions through electron transfer from intracellular or extracellular biomolecular donors to Ag^+^ ions, resulting in their reduction to elemental silver (Ag^0^). Importantly, these biomolecules not only initiate reduction but also play essential roles in stabilizing the resulting nanoparticles by forming capping layers that prevent aggregation and modulate physicochemical properties such as size, morphology, and surface charge [27,28,34,35].

The relative contribution of each biomolecular class is strongly influenced by fungal species, growth phase, and environmental conditions, underscoring the absence of a universal mechanism and highlighting the need for a system-level understanding of fungal-mediated nanobiosynthesis.

### 4.1. Enzymes as Primary Reducing Agents

Enzymes represent one of the most extensively investigated classes of biomolecular drivers in fungal-mediated AgNP biosynthesis. Among them, nitrate reductase has been consistently identified as a key enzyme involved in extracellular silver ion reduction, particularly in NADH or NADPH-dependent forms [19]. This enzyme catalyzes the reduction of nitrate to nitrite while simultaneously facilitating electron transfer to Ag^+^ ions.

Experimental evidence demonstrates that nitrate reductase activity is highly dependent on growth conditions and physiological state. In *Fusarium oxysporum*, enzyme activity increases from 37 nmol nitrite mL^−1^ h^−1^ during early growth phases to 259 nmol mL^−1^ h^−1^ in the stationary phase [27]. Culture medium composition and environmental factors such as light exposure significantly influence enzyme output; for example, cultivation in MGYP medium under light conditions resulted in 151 nmol nitrite mL^−1^ h^−1^ compared to only 53 nmol mL^−1^ h^−1^ under dark conditions, while nitrate-enriched media further increased activity to 220 nmol mL^−1^ h^−1^ [27].

Additional studies have reported notable physicochemical properties of nitrate reductase, including thermostability up to 100 °C with a half-life of 1.55 h while retaining approximately 70% of its initial activity [16]. Mechanistically, some reports propose that nitrate reductase exists in a phosphorylated inactive form that becomes activated in the presence of Ag^+^ ions, accompanied by the release of inorganic phosphate—an event supported by the appearance of characteristic UV–Vis peaks at 430 nm (AgNP formation) and 360 nm (phosphate release) [28]. Notably, this activation mechanism appears to be species-specific, occurring in *Aspergillus flavus*, *A. niger*, and *A. terreus* but not in *Fusarium oxysporum* or *Trichoderma reesei* [28].

Beyond nitrate reductase, additional oxidoreductive enzymes contribute to Ag^+^ reduction. FAD-dependent oxidoreductases, identified in *Aspergillus oryzae* and *Fusarium oxysporum*, have been reported with molecular weights of 37.4 kDa and 60.4 kDa and utilize FADH_2_ as an electron donor [36]. Cytochrome-mediated electron transport systems have also been detected, with proteins from Chaetomium globosum showing similarity to cytochrome P450 and ubiquinol–cytochrome c reductase systems, suggesting the involvement of respiratory redox chains [37].

Other enzyme classes implicated in biosynthesis of AgNPs include peroxidases, chitinases, and glycoside hydrolases. Peroxidases identified in *Aspergillus flavus* function as potent reductants through reactive surface groups rather than strictly catalytic activity [38,39]. Chitinase from *Duddingtonia flagrans* (34 kDa, optimal pH 8–10 at 60 °C) has been shown to act as both a reducing and a capping agent at a concentration of 0.22 U mL^−1^ [40]. Proteolytic enzymes such as aspartyl proteinases, serine carboxypeptidases, and aminopeptidases may indirectly contribute by generating peptide fragments that stabilize nanoparticles [41]. Together, these findings suggest that enzyme-mediated reduction is highly context-dependent and influenced by both species-specific expression profiles and environmental conditions, further complicating mechanistic interpretation.

### 4.2. Proteins: Dual Roles in Reduction and Capping

Proteins constitute a central functional component of fungal secretomes and exhibit dual roles in AgNP biosynthesis, functioning both as reducing agents and as capping/stabilizing molecules. Several proteins have been directly associated with silver ion reduction. These include nitrate reductase in *Fusarium* and *Aspergillus* spp. [42], FAD-dependent oxidoreductases in *Aspergillus oryzae* [43], 1,4-*α*-glucosidase in *Fusarium solani* [44], and laccase in *Trametes versicolor* and *Alternaria arborescens* [45]. Additionally, low-molecular-weight proteins such as the 15 kDa protein in *Neurospora intermedia* and the 32 kDa protein in *Aspergillus flavus* have been proposed as active reducing agents [7,46]. At the same time, numerous proteins primarily function as capping agents, forming stabilizing layers around nanoparticles. Examples include the 23 kDa protein in *N. intermedia*, the 35 kDa protein in *A. flavus*, and the 85 kDa protein in *Macrophomina phaseolina* [7,46,47]. Actin has been identified as a major capping protein in *Penicillium aculeatum*, while glucoamylase in *Thermomyces lanuginosus* contributes to nanoparticle stability at elevated temperatures [48,49]. Proteins bind to nanoparticle surfaces through multiple functional groups present in amino acid residues, including amino (-NH_2_), hydroxyl (-OH), carboxyl (-COOH), and thiol (-SH) groups, as well as the aromatic rings of phenylalanine, tyrosine, tryptophan, and histidine residues. Computational studies have shown that the *π*-electron cloud of aromatic rings interacts with metal surfaces through a combination of dative and dispersive non-covalent interactions, with the aromatic ring preferring a flat-on orientation relative to the nanoparticle surface that maximizes electron overlap [50]. Proteins bind to nanoparticle surfaces through functional groups such as amino, hydroxyl, carboxyl, and thiol groups. In *Aspergillus tubingensis*, eight secreted proteins including glycoamylase, acid phosphatase, and glucanosyltransferase were shown to form multilayer capping structures via cysteine-mediated S–Ag interactions [34]. Such multilayered protein coronas significantly influence nanoparticle stability, dispersion, and reactivity. Proteins from *Saccharomyces boulardii* and *Trichoderma harzianum* have been identified as both reducing and stabilizing agents, further supporting their multifunctional role in fungal nanoparticle biosynthesis [51,52]. Individual amino acids have been shown to reduce Ag^+^ and direct nanoparticle formation under purely chemical conditions (findings not conducted in fungal systems, but mechanistically relevant to mycosynthesis). Cysteine, bearing a thiol side chain with high affinity for silver, simultaneously acts as a reductant and a surface capping ligand through stable Ag-S bond formation [11]. Tryptophan reduces Ag^+^ under alkaline conditions via the oxidation of its indole ring, likely through the kynurenine pathway, with the oxidation products remaining adsorbed on the particle surface. Tyrosine, with its oxidizable phenolic hydroxyl group, has been similarly implicated in Ag^+^ and Au^3+^ reduction [12]. Although none of these studies were performed in fungal contexts, cysteine, tryptophan, and tyrosine are ubiquitous proteinogenic amino acids present in fungal secreted proteins and peptides (suggesting they may contribute to the reductive capacity of cell-free fungal filtrates during mycosynthesis) [53].

### 4.3. Polysaccharides and Saccharides in Reduction and Stabilization

Polysaccharides, particularly extracellular polymeric substances (EPSs), are increasingly recognized as important contributors to fungal-mediated AgNP biosynthesis. These macromolecules participate in both reduction and stabilization processes through their abundant hydroxyl and carbonyl functional groups.

Detailed structural studies of EPS from *Cordyceps sinensis* Cs-HK1 have identified multiple fractions (P0.5, P2.0, and P5.0) differing in polysaccharide-to-protein ratios and molecular weights. Specifically, P0.5 contained 93% polysaccharide and 7% protein (~1022 kDa), P2.0 contained 69% polysaccharide and 31% protein (~85.16 kDa), and P5.0 contained 54% polysaccharide and 46% protein (~21.67 kDa), with all fractions primarily composed of mannose, glucose, and galactose [54]. Similar heteroglycan compositions have been reported in related studies [55].

Exopolysaccharides from *Aspergillus japonicus* at concentrations of 0.81 ± 0.3 mg mL^−^^1^, along with reducing sugars (0.54 ± 0.4 mg mL^−^^1^), have been shown to contribute to nanoparticle formation through O–H and C–OH functional groups [35]. Glucans from *Pleurotus* spp. act as both reducing and capping agents while also modulating AgNP toxicity and enhancing antimicrobial activity [56]. Additional polysaccharide-based stabilizers include chitosan, which binds to AgNPs through amide linkages [57], and carboxymethylcellulose (CMC), which enhances colloidal stability via carboxyl group interactions. In one study, CMC-stabilized AgNPs exhibited long-term stability exceeding one year, with zeta potential values shifting from −25.0 mV to −27.5 mV [58].

Despite this evidence, the mechanistic role of polysaccharides remains insufficiently explored, with most studies relying on indirect spectroscopic characterization rather than direct biochemical validation.

### 4.4. Secondary Metabolites as Redox Mediators and Capping Agents

Secondary metabolites represent a chemically diverse and mechanistically significant class of biomolecules involved in fungal-mediated AgNP biosynthesis. These compounds including phenolics, flavonoids, terpenoids, and alkaloids can act as both reducing agents and stabilizers.

A mechanistic framework proposed for *Trichoderma* spp. suggests that extracellular metabolites, particularly phenolics and flavonoids, reduce Ag^+^ ions while simultaneously acting as capping agents during nanoparticle nucleation and growth [59,60]. This dual functionality links reduction and stabilization processes and distinguishes metabolite-driven mechanisms from enzyme-dependent pathways.

Phenolic compounds such as syringic acid, sinapic acid, rosmarinic acid, gallic acid, quercetin, and caffeic acid identified in *Agaricus bisporus* extracts have been shown to donate electrons via hydroxyl groups while coordinating with nanoparticle surfaces through carboxylate groups [29]. Additional compounds, including epicatechin-6-glucoside in *Ganoderma lucidum* [30] and puerarin and genistein in *Trichoderma harzianum* [31], further demonstrate the chemical diversity of metabolite-driven reduction.

Rapid nanoparticle formation has been observed in systems containing tannins, flavonoids, and β-carotenes from *Nigrospora* spp., with visible color change occurring within 10 min at room temperature [32]. FTIR analysis across multiple studies has confirmed the presence of phenolic functional groups on nanoparticle surfaces and indicated oxidation of hydroxyl groups during silver ion reduction [33].

**Figure 2:**
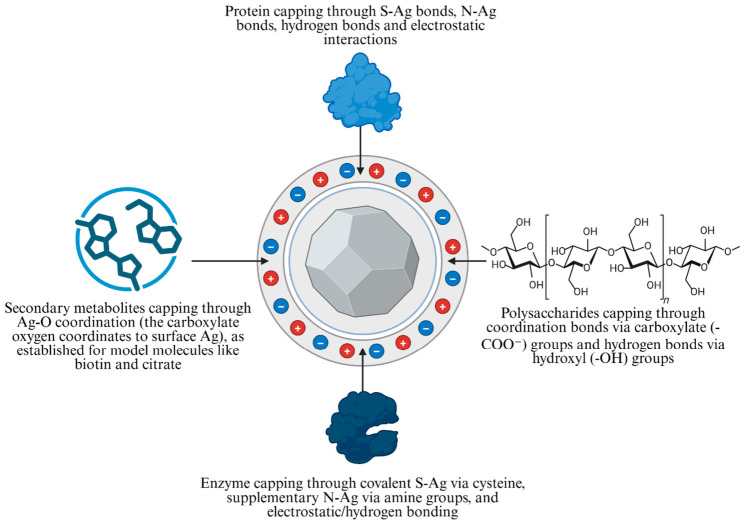
Schematics of enzyme, protein, polysaccharides and secondary metabolites capping on silver nanoparticle. Created in BioRender. Vorkapić, M. (2026) https://BioRender.com/gqvvtej (accessed on 8 June 2026).

Terpenoids and related compounds also contribute to reduction and stabilization. β-carotenes, triterpenoids, and aliphatic hydrocarbons detected in *Isaria fumosorosea* and *Talaromyces purpureogenus* have been associated with AgNP formation, as confirmed by FTIR and GC–MS analyses [31,32,61,62,63]. Alkaloids have been reported in a limited number of studies and their roles remain poorly characterized. While amine-containing compounds may contribute to nanoparticle stabilization through amide linkages, direct mechanistic evidence is lacking [64,65]. Overall, secondary metabolites likely act as a collective redox system, rather than as individual isolated agents, highlighting the importance of metabolomic complexity in fungal-mediated nanoparticle synthesis. The identification of individual biomolecular contributors provides a foundation for understanding how silver ion reduction occurs; however, these components operate within broader mechanistic pathways that remain incompletely resolved. A schematic representation of the combined roles of enzymes, proteins, polysaccharides, and secondary metabolites in nanoparticle capping and stabilization is shown in Figure 2. L-DOPA and its polymer poly(L-DOPA) represent a structurally distinct but mechanistically related class of reductants. The catechol moiety undergoes oxidation to the corresponding quinone, releasing electrons that reduce Ag^+^ to Ag^0^, while the resulting polydopamine layer simultaneously stabilizes the nanoparticle surface without any additional capping agent. Turan et al. demonstrated this dual role in a non-fungal chemical system, where poly(L-DOPA) drove Ag^+^ reduction onto gold nanorod cores to yield Au@Ag core–shell nanostructures with tunable morphology [66]. Reported polydopamine-mediated confirmed the same principle, with the PDOP layer acting as both reductant and interface [67]. Although these studies were conducted under purely chemical conditions with no involvement of fungi, L-DOPA is a known precursor in fungal melanin biosynthesis (raising the possibility that catechol-type metabolites secreted by melanogenic fungi may contribute analogously to Ag^+^ reduction during mycosynthesis).

## 5. Mechanistic Pathways of Silver Ion Reduction

While individual biomolecules contribute to AgNP biosynthesis, their roles converge in several proposed mechanistic pathways that describe the reduction of Ag^+^ to elemental silver (Ag^0^). These mechanisms are not mutually exclusive and are likely to operate simultaneously or synergistically, depending on fungal species, secretome composition, and environmental conditions.

Current mechanistic models primarily include enzyme-driven reduction, reactive oxygen species-mediated pathways, and metabolite-driven redox processes, each supported by varying degrees of experimental evidence. However, most proposed mechanisms remain based on indirect observations, and their relative contributions to nanoparticle formation are not yet fully resolved. The following sections summarize the principal mechanistic frameworks proposed for fungal-mediated silver ion reduction.

### 5.1. Nitrate Reductase-Dependent Mechanism

A widely proposed mechanism for fungal-mediated AgNP biosynthesis involves nitrate reductase as the primary reducing enzyme. In this model, nitrate reductase transfers electrons from NADH or NADPH to silver ions, reducing Ag^+^ to elemental silver [19,21,22,23,24].

**Figure 3:**
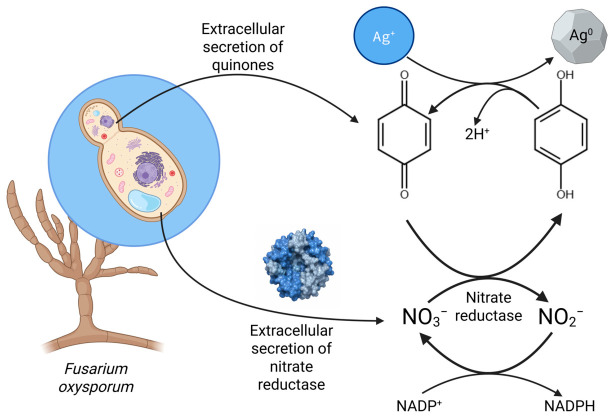
Proposed mechanism of silver ion reduction via nitrate-dependent reductase and a shuttle quinone extracellular process. Created in BioRender. Vorkapić, M. (2026) https://BioRender.com/fu0qczi (accessed on 8 June 2026).

In *Fusarium oxysporum*, this process has been described as part of a coupled system involving extracellular quinone redox mediators that function as electron shuttles between the enzyme and silver ions [68]. This mechanism, involving electron transfer from NAD(P)H through nitrate reductase and quinone mediators to Ag^+^ ions, is schematically illustrated in Figure 3. The presence of nitrite as a reaction product and the observed correlation between enzyme activity and nanoparticle formation provide indirect support for this mechanism [21]. Additional studies have suggested that nitrate reductase may undergo activation through phosphorylation–dephosphorylation processes in certain fungal species, further indicating species-specific regulatory mechanisms [28]. However, direct experimental validation of this pathway remains limited, and its relative contribution compared to alternative mechanisms is still under debate.

### 5.2. Superoxide-Mediated Reduction

An alternative and potentially complementary mechanism involves the generation of reactive oxygen species (ROS), particularly superoxide radicals ($O_{2}^{-}$), as reducing agents.

**Figure 4:**
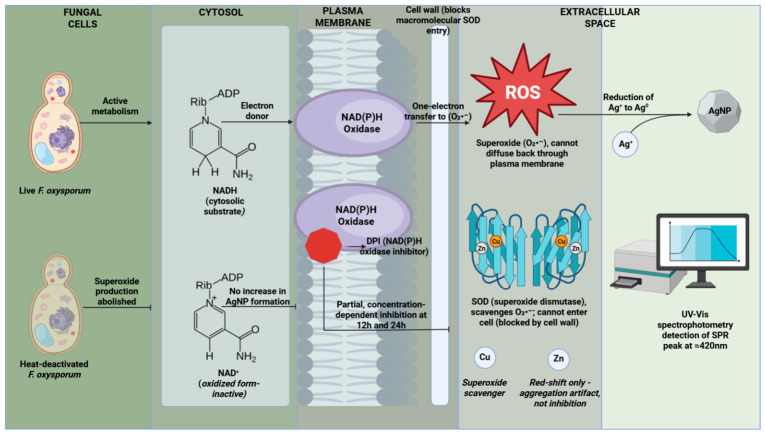
Proposed mechanism of extracellular AgNP biosynthesis by *Fusarium oxysporum*, involving NAD(P)H oxidase-mediated generation of superoxide radicals (ROS), which reduce Ag^+^ ions to Ag^0^ and promote nanoparticle formation. The re-quirement for active metabolism is confirmed by inhibition and scavenging experiments as well as the absence of AgNP formation in heat-inactivated cells. Created in BioRender. Vorkapić, M. (2026) https://BioRender.com/4nsbztg (accessed on 8 June 2026).

In this model, extracellular superoxide produced by fungal metabolism directly reduces Ag^+^ to Ag^0^ in solution [39]. The proposed superoxide-driven reduction mechanism is illustrated in Figure 4. This mechanism contrasts with enzyme-centered models by emphasizing reactive metabolites rather than specific enzymatic pathways.

Experimental evidence for this pathway includes studies demonstrating superoxide production during fungal growth and its correlation with AgNP formation. However, similar to the nitrate reductase model, the evidence remains largely indirect and does not establish a definitive causal relationship between superoxide generation and nanoparticle formation. Furthermore, the relative contribution of ROS-mediated reduction compared to enzyme-driven pathways remains unresolved and may vary significantly between fungal species. Even though superoxide exhibites silver reduction properties its role should not be taken as chemically unambiguous. The same pH window that sustains O_2_·^−^ long enough to reduce Ag^+^ also drives competing dismutation to H_2_O_2_, which in turn re-oxidizes nascent AgNPs through a cyclic charge–discharge mechanism [69]. Beyond superoxide, extracellular proteins with oxidoreductase function (including nitrate reductases, peroxidases, and an array of as-yet-unidentified reducing metabolites) contribute independently to Ag^+^ reduction and nanoparticle capping, as documented across multiple fungal systems [70]. The honest summary is that superoxide represents one documented electron-transfer pathway among several operating in parallel; its relative contribution depends on organism, growth conditions, pH, and the instantaneous ROS balance in the extracellular milieu, and has not been quantitatively resolved in any system.

### 5.3. Metabolite-Driven Reduction Pathways

A third mechanistic framework emphasizes the role of small, diffusible metabolites, particularly phenolics, flavonoids, and other redox-active compounds as primary drivers of silver ion reduction.

**Figure 5:**
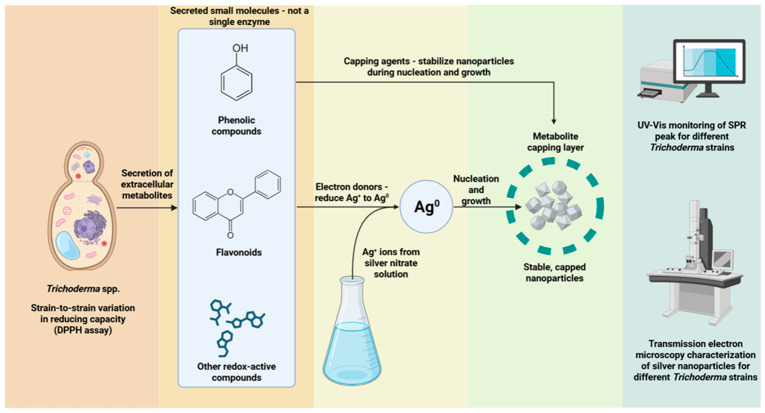
Proposed metabolite-mediated mechanism of silver nanoparticle biosynthesis by *Trichoderma* spp. Extracellular phenolic compounds, flavonoids, and other redox-active metabolites reduce Ag^+^ ions to Ag^0^, promoting nanoparticle nuclea-tion and growth, while simultaneously acting as capping agents that stabilize the resulting AgNPs. Created in BioRender. Vorkapić, M. (2026) https://BioRender.com/obgq87n (accessed on 8 June 2026).

In this model, extracellular metabolites reduce Ag^+^ through direct electron donation while simultaneously acting as capping agents during nanoparticle nucleation and growth [60]. This dual-function mechanism, linking reduction and stabilization by small redox-active molecules, is illustrated in Figure 5.

Functional group analysis suggests that hydroxyl groups participate in redox reactions, while carboxyl and amine groups facilitate surface binding and stabilization [29,33]. Unlike enzyme-driven mechanisms, metabolite-driven pathways do not rely on catalytic specificity and therefore may operate under a broader range of physicochemical conditions. This mechanism is particularly consistent with studies reporting rapid nanoparticle formation and strong correlations between metabolite profiles and synthesis efficiency. However, due to the complexity of fungal metabolome, isolating the effects of individual compounds remains a major challenge, and mechanistic assignments are often based on indirect evidence.

### 5.4. Critical Considerations

Representative mechanistic studies highlighting fungal species, biomolecular drivers, and analytical approaches are summarized in Table 1.

The taxonomic scope of mechanistically informative studies remains severely restricted. The overwhelming majority of primary studies focus on filamentous ascomycetes, principally *Fusarium* spp., *Aspergillus* spp., *Penicillium* spp., and *Trichoderma* spp., with limited representation of Basidiomycota (largely restricted to mushroom-forming species studied with partial characterization [71,72,73,74]), yeasts (represented by only a small number of studies with limited mechanistic depth [75,76,77]), and an almost complete absence of non-ascomycete filamentous lineages such as Mucoromycota, Chytridiomycota, or lichenized fungi. Consequently, mechanistic conclusions derived predominantly from a small number of ascomycete genera may not reflect the full diversity of biomolecular strategies employed across the fungal kingdom.

Even within well-studied genera, strain-level variation in biomolecular composition results in significantly different nanoparticle properties and yields [36,77]. However, comparative proteomic or metabolomic studies across strains remain rare, and no study to date has systematically mapped the relationship between secretome composition and nanoparticle formation efficiency across a phylogenetically diverse fungal panel.

The roles of saccharides and polysaccharides as reducing or capping agents remain comparatively underexplored relative to proteins and enzymes. Only a limited number of studies provide evidence for polysaccharide participation, most notably the *Cordyceps sinensis* EPS study [54] and yeast-derived polymeric carbohydrate systems, and even these rely primarily on indirect spectroscopic (FTIR-based) evidence. Moreover, the potential role of cell wall polysaccharides as nucleation templates or electrostatic ion-trapping scaffolds prior to extracellular reduction remains entirely uninvestigated. From a methodological perspective, the dominance of crude cell-free filtrate experiments combined with ensemble spectroscopic readouts has significantly limited mechanistic resolution. Approaches capable of establishing causality, such as fractionation, reconstitution experiments, enzyme inhibition studies, and genetic knockouts, are largely absent. As a result, most mechanistic interpretations remain at the level of correlation rather than demonstrating clear necessity or sufficiency of specific biomolecular classes [78,79,80]. Addressing these limitations will be essential for transitioning fungal-mediated AgNP biosynthesis from descriptive studies toward predictive and mechanistically grounded nanobiotechnology.

## 6. Factors Affecting Fungal AgNP Biosynthesis

Both intracellular and extracellular mycosynthesis share the same core redox chemistry bioreduction of dissolved Ag^+^ to metallic Ag^0^, as well as the fundamental materials science processes of nucleation, growth, and biomolecule-mediated stabilization (capping). However, they differ primarily in the location of nanoparticle formation and in downstream recovery processes [18,78,79,80,81].

Across fungal systems, synthesis outcomes are commonly tuned by adjusting key parameters such as temperature, pH, silver precursor concentration, biomass loading, and incubation time, although optimal conditions vary depending on fungal species and experimental design [18].

The most significant distinction lies in whether intact fungal biomass is present during nanoparticle synthesis or whether a cell-free secretome serves as the reaction medium [18]. In intracellular synthesis, the silver precursor is internalized, and nanoparticle formation occurs within or just beneath the cell wall surface. This is supported by thin-section electron microscopy showing particles localized below the cell wall, consistent with reduction mediated by enzymes associated with the cell wall membrane [82].

**Figure 6:**
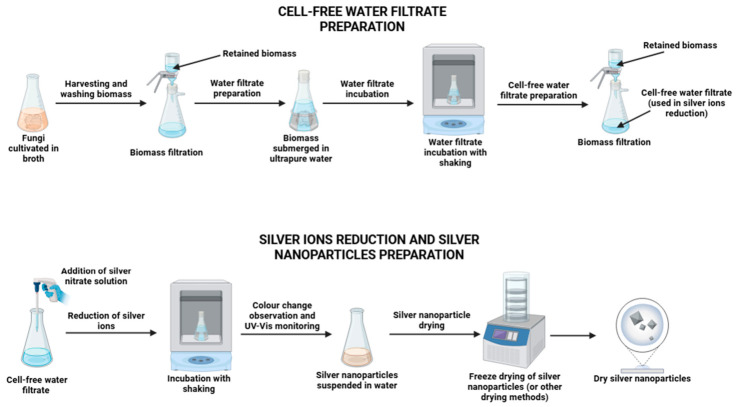
Schematic overview of laboratory protocol for extracellular AgNP biosynthesis. Created in BioRender. Vorkapić, M. (2026) https://BioRender.com/60uzx5c (accessed on 8 June 2026).

In contrast, extracellular synthesis takes place in the aqueous filtrate, where secreted enzymes and metabolites interact directly with Ag^+^ ions in solution. This approach is widely considered advantageous because nanoparticle formation occurs in solution, greatly simplifying downstream processing compared to intracellular routes (Figure 6) [18,83].

A second key difference concerns enzyme localization and the accessibility of reducing biomolecules [82]. Intracellular synthesis is associated with membrane-bound or cell wall-associated enzymes, along with initial metal ion binding at the cell surface. By contrast, extracellular synthesis relies on enzymes and metabolites present in the filtrate, including nitrate reductase-like activities and NADH-dependent systems that are frequently proposed as major contributors to biogenic reduction [81,82,83].

A third distinction relates to the capping environment and the composition of the nanoparticle-associated organic shell [79]. Extracellular studies provide direct evidence that proteins and metabolite-derived functional groups cap and stabilize nanoparticles, as supported by TEM observations of interparticle separation and FTIR signals corresponding to protein and organic functional groups [80]. In contrast, intracellular systems are primarily characterized by nanoparticle localization and enzyme-mediated reduction near the cell wall membrane, while the identity of intracellular capping agents remains less clearly defined [82].

Finally, the two synthesis routes differ substantially in recovery requirements, which has important implications for scalability and reproducibility [84]. Intracellular synthesis requires biomass disruption followed by chemical treatment and separation techniques such as centrifugation or filtration to release nanoparticles. In contrast, extracellular synthesis enables more straightforward recovery through purification of colloidal dispersion using methods such as centrifugation, washing, filtration, dialysis, or ultracentrifugation, depending on the desired application [18].

### 6.1. Silver Precursor Concentration

Most studies report optimal silver nitrate concentrations in the range of 0.5–2 mM, often exhibiting clear saturation behavior. However, the effect of silver precursor concentration is complex and non-linear across different fungal systems. Generally, increasing silver nitrate concentration enhances AgNP yield up to an optimal point, beyond which nanoparticle quality deteriorates due to aggregation or reduced stability.

In *Aspergillus sydowii*, absorbance (at 420 nm) reached 0.37 at 0.5 mM silver nitrate, increased to approximately 0.75–0.78 at 1.5 mM, and subsequently decreased to 0.58 at 2.0 mM and 0.56 at 2.5 mM [85]. This trend illustrates a clear optimal concentration window with reduced efficiency at higher concentrations. A similar pattern was observed in *Aspergillus terreus* ITCC 9932.15, where the surface plasmon resonance (SPR) peak intensity increased from negligible values at 0.25 mM to approximately 1.0–1.1 at 0.5–1.0 mM. At higher concentrations (1.5–2.0 mM), peak broadening was observed despite increased absorbance, indicating the onset of nanoparticle aggregation [86].

The optimal silver nitrate concentration is strongly species-dependent. For instance, *Penicillium purpurogenum* CATMC-AH-1 exhibited a positive correlation between silver nitrate concentration and UV–Vis absorbance, with values of 1.8 at 1 mM, 2.0 at 1.5 mM, and 2.1 at 2 mM [87]. In contrast, *Aspergillus niger* cell-free filtrate showed an inverse relationship, with the highest absorbance (~1.188) at 1 mM and a decrease to ~0.546 at 5 mM, suggesting enzyme–substrate saturation at concentrations above ~1 mM [88].

Silver nitrate concentration also influences nanoparticle size, often displaying opposing trends depending on the concentration range. In *Aspergillus oryzae* MTCC 1846, particle size decreased from 17.06 ± 5.78 nm at 1 mM to 7.22 ± 3.07 nm at 8 mM, followed by a sharp increase to 45.93 ± 22.81 nm at 9 mM and 62.12 ± 31.5 nm at 10 mM, indicating a U-shaped relationship. This behavior was attributed to the availability of functional groups from secreted proteins, which promote rapid nucleation and inhibit particle growth at moderate concentrations but become limiting at higher concentrations, leading to aggregation [19]. Similarly, *Talaromyces funiculosus* produced a symmetric SPR peak at 422.5 nm at 1 mM with particle sizes of ~34 nm, whereas higher concentrations (4 mM) resulted in increased absorbance but also peak broadening and precipitation due to insufficient capping capacity [89]. The threshold for nanoparticle formation also varies between species. While many systems require concentrations between 0.5 and 1 mM, *Phellinus adamantinus* showed no nanoparticle formation at 0.5 mM but produced stable particles at 1 mM [90]. In contrast, *Penicillium polonicum* PG21 exhibited detectable synthesis at 5 mM, with an optimal concentration reported at 80 mM [91]. Upper concentration limits also differ significantly across studies. For example, *Penicillium oxalicum* GRS-1 showed a decline in SPR intensity above ~1.5–2.0 mM, while *Saccharomyces ellipsoideus* BSU-XR1 exhibited inhibition at concentrations above 3.0 mM [92]. Collectively, these findings demonstrate that silver precursor concentration is a critical but highly system-dependent parameter. While moderate concentrations generally promote efficient nucleation and controlled growth, excessive precursor levels can disrupt the balance between reduction and stabilization processes, leading to aggregation and reduced nanoparticle quality.

#### Critical Considerations

The most consequential limitation in current research is the near-universal reliance on one-factor-at-a-time experimental designs, which preclude the identification of interactions between AgNO_3_ concentration and co-varying parameters such as pH, temperature, biomass loading, and filtrate composition [88,93,94]. This methodological constraint implies that reported “optimal” concentrations are conditional upon fixed levels of other variables and cannot be generalized across fungal systems. Consequently, it is not currently possible to reliably predict how changes in a single parameter (e.g., shifting pH from 6 to 9) would influence the optimal AgNO_3_ concentration for a given species. Although several studies have reported concentration-dependent saturation or aggregation effects [88,95,96], the lack of factorial or response surface methodology (RSM) designs prevents systematic characterization of the underlying boundary conditions and mechanistic drivers.

A second major gap concerns the narrow and inconsistent concentration ranges evaluated across studies. Many investigations examined only one to three AgNO_3_ concentrations [46], while studies exploring broader ranges were typically limited to 1–5 mM [93]. Li et al. (2021) represents a notable exception, reporting concentrations up to 0.8 mol/L [35]; however, these values are difficult to reconcile with the majority of the existing literature, and no clear rationale for concentration selection was provided. The absence of systematic dose–response characterization—particularly in sub-millimolar and supra-5 mM regimes—means that key thresholds for enzyme saturation, substrate inhibition, and nanoparticle aggregation remain poorly defined. Furthermore, the mechanistic basis for optimal concentration selection remains largely unexplored. Only a limited number of studies have invoked enzyme–substrate kinetics [88] or used indirect markers such as inorganic phosphate release as proxies for nitrate reductase activity [87], and none have quantified kinetic parameters (e.g., K_m_, V_max_) as a function of AgNO_3_ concentration. This mechanistic gap limits the rational design of biosynthesis protocols and constrains extrapolation to previously unstudied fungal species.

A third limitation lies in the restricted scope of optimization criteria and the lack of application-oriented performance metrics. Most studies define “optimal” concentration based solely on UV–Vis absorbance (as a proxy for yield) [95] or particle size [87,97,98], without simultaneous consideration of colloidal stability (e.g., zeta potential), monodispersity, or functional performance (e.g., antimicrobial activity or MIC values). Even in studies that evaluated antibacterial activity, testing was typically conducted at a single “optimized” concentration rather than across a full concentration gradient [35,95]. As a result, multi-objective optimization, balancing yield, size, stability, and bioactivity, remains largely unexplored. Additionally, key parameters such as long-term stability, batch-to-batch reproducibility, and scalability as functions of AgNO_3_ concentration are rarely addressed.

Finally, although a relatively broad range of fungal species has been studied, direct comparative analyses remain scarce. Few studies have evaluated multiple species under standardized conditions; for example, Mekawey and Helmy (2017) screened six fungal species but applied only a single AgNO_3_ concentration [99]. Without systematic, side-by-side comparisons, it remains unclear whether observed trends are species-specific or represent generalizable principles of fungal-mediated AgNP biosynthesis.

### 6.2. Incubation Time

Incubation time generally exerts a positive effect on AgNP biosynthesis, with most studies reporting gradual increases in nanoparticle formation followed by plateau or saturation behavior. The onset of nanoparticle formation varies widely between systems, ranging from minutes to several days depending on reaction conditions. For example, *Penicillium polonicum* synthesizes AgNPs within approximately 60 min under optimized conditions (pH 10 and a 1:4 ratio of silver nitrate to culture filtrate) [100]. Similarly, *Aspergillus japonicus* PJ01 has been reported to reduce silver ions within 1 min using high precursor concentrations (0.8 M AgNO_3_) and alkaline conditions (1.5 M NaOH) at 30 °C [26]. However, under typical ambient conditions, nanoparticle formation generally requires 24–72 h [101]. Time-dependent increases in nanoparticle formation are consistently observed across fungal systems. In *Alternaria alternata*, absorbance increased from negligible values at 0 h to approximately 0.45 at 24 h, 0.6 at 48 h, 0.8 at 72 h, and ~0.9 at 96–108 h, with a maximum reached at 120 h [102]. A similar trend was reported for *Trichoderma virens* HZA14, where absorbance increased from 1.2 at 24 h to 1.4 at 48 h, 1.6 at 72 h, 1.8 at 96 h, and 1.9–2.0 at 120 h [103].

Most studies report completion of nanoparticle formation within 48–144 h. In *Trichoderma reesei*, AgNP synthesis reached a plateau after approximately 145 h under optimized culture conditions (Medium 3 with 10 mM AgNO_3_) [21]. Similarly, *Neopestalotiopsis clavispora* reached peak absorbance (1.499) at 64 h, followed by stabilization at ~1.498 at 72 h [104]. Prolonged incubation beyond the plateau phase may lead to nanoparticle aggregation or growth. For instance, *Aspergillus niger* showed minimal changes in SPR intensity between 120 h and 2 months, but a shift in peak position from ~420–425 nm to ~440 nm occurred after extended incubation indicated particle growth [88]. The effect of incubation time is strongly influenced by other parameters, particularly pH and temperature. At pH 9, *Fusarium* 4F1 and *Trichoderma* TrS exhibited gradual increases in absorbance up to 72 h without evidence of aggregation. In contrast, at pH 7–8, prolonged incubation (9–10 days) resulted in visible aggregation, characterized by darkening of the solution, sediment formation, and red-shifting of SPR peaks [105].

Collectively, these findings indicate that incubation time is a key kinetic parameter governing nanoparticle nucleation and growth. While extended reaction times generally enhance nanoparticle formation, excessive incubation can promote secondary growth processes and aggregation, highlighting the importance of identifying system-specific optimal time windows.

#### Critical Considerations

The most critical gap across this body of evidence is the near-complete absence of rigorous kinetic modeling and mechanistic investigation of how incubation time governs nucleation, growth, and stabilization of AgNPs during fungal biosynthesis. While several studies have performed time-course monitoring using UV–visible spectroscopy [21,85,105], none have applied formal kinetic models (e.g., Avrami or Finke–Watzky models) to quantify reaction rate constants or distinguish between nucleation and growth phases. Only a single study has explicitly linked incubation time to nitrate reductase activity and fungal growth phase, thereby providing a mechanistic basis for the observed optimum. In contrast, the majority of studies treat incubation time as an empirical variable without addressing the underlying biochemical dynamics. As a result, optimal incubation times identified in one system cannot be rationally predicted or transferred to other fungal systems, significantly limiting the translational value of the existing evidence. Furthermore, although Quester et al. [106] highlighted that the mechanisms of fungal-mediated AgNP biosynthesis remain poorly understood, and Gemishev et al. [21] reported a biotransformation plateau beyond 145 h without kinetic analysis, no study has systematically addressed this knowledge gap. Most studies rely on UV–visible absorbance intensity as the primary optimization criterion [85,95,102], which serves only as a proxy for nanoparticle concentration and provides limited insight into particle size distribution, colloidal stability, or functional performance over time. Notably, no study has systematically examined how variation in incubation time affects protein corona composition, surface charge evolution, or functional properties such as antimicrobial or cytotoxic activity across a controlled time series. This limitation is particularly significant, as nanoparticles optimized for maximum yield may not exhibit optimal size uniformity, biological activity, or long-term stability—parameters that are critical for biomedical and agricultural applications.

Additionally, no study has evaluated the economic and energetic trade-offs between rapid, high-temperature synthesis routes [26] and slower, ambient-condition protocols [95]. Such analyses are essential for assessing scalability and industrial feasibility, yet they remain entirely absent from the current literature.

### 6.3. Culture Media Composition

Culture medium composition plays a critical role in fungal-mediated AgNP biosynthesis by modulating the profile of secreted reducing and capping biomolecules.

In *Aspergillus niger* cell-free filtrate systems, Potato Dextrose Broth (PDB) yielded the highest UV–Vis absorbance (~1.832), compared to MYPG (~1.67), Czapek broth (~1.231), Sabouraud dextrose broth (~0.994), and GPYB (~0.647) [88]. However, when comparing PDB and Sabouraud Dextrose Broth (SDB) across different *A. niger* isolates, SDB produced higher AgNP yields (absorbance ~0.532 vs. ~0.348 at 450 nm), which was attributed to increased nitrogen availability enhancing nitrate reductase production [107].

Specialized enzyme-inducing media can further optimize nanoparticle yield and monodispersity. In *Aspergillus oryzae* MTCC 3107, an amylase production medium (APM containing 1% soluble starch as an inducer) generated a stronger and sharper SPR peak at ~420 nm (~0.78 absorbance compared to ~0.6 in PDB). Additionally, this medium produced more uniform and spherical nanoparticles (~40 nm), whereas PDB resulted in polydisperse particle distributions [108]. Systematic comparisons of multiple media formulations have demonstrated strong effects on both synthesis kinetics and nanoparticle properties. In one study, five media variants were evaluated, with Medium 3 (comprising base salts, 2% glucose, and 0.1% corn steep liquor) producing the highest yield, fastest reduction rate, and smallest, nearly monodisperse nanoparticles (2–6 nm). Media containing peptone (Medium 4) and casamino acids (Medium 5) accelerated initial reduction (SPR peak appearance at ~24 h) but resulted in larger particles (4–11 nm). In contrast, yeast extract-based medium (Medium 2) exhibited a slower onset of nanoparticle formation (~72 h), while the base medium alone (Medium 1) showed the slowest kinetics (~96 h) and the lowest overall yield [108,109].

Variations in culture media also directly impact extracellular metabolite profiles. FTIR analysis of AgNPs synthesized using five different *Trichoderma reesei* media revealed distinct capping signatures, with Medium 3 providing the most balanced combination of reducing and stabilizing biomolecules [110]. Similarly, in *Fusarium oxysporum*, a modified medium enriched with nitrate and elevated carbon content produced the highest productivity, with UV–Vis peak absorbance reaching ~3.6–3.8 at 72 h compared to ~3.2–3.4 in standard MGYP medium [27].

Collectively, these findings demonstrate that culture medium composition is a key determinant of both nanoparticle formation kinetics and physicochemical properties. By controlling nutrient availability and inducing specific metabolic pathways, media composition directly shapes the composition of the fungal secretome and, consequently, the efficiency and quality of AgNP biosynthesis.

#### Critical Considerations

The most consequential gap across this body of literature is the near-complete absence of systematic, head-to-head comparisons of culture media under otherwise controlled conditions. Only a limited number of studies have addressed this issue. Alamilla-Martínez et al. compared sucrose and Czapek media [87], Gemishev et al. evaluated five media variants for *T. reesei* [110], Al-Hamadani and Kareem tested five media formulations for *A. niger* [88], and Gupta and Saxena contrasted PDB with amylase production media for *A. oryzae* [108]. While these studies clearly demonstrate that medium composition directly influences AgNP size distribution, yield, and monodispersity, none propose generalizable standardization frameworks applicable across different fungal systems.

The remaining studies overwhelmingly rely on a single medium—most commonly PDB [100,101,102], MGYP [86,87,88,89], SDB [101,107], or custom mineral salt formulations [104,109] without providing justification for medium selection or evaluating alternative conditions. This fragmented approach introduces a significant source of variability, as reported “optimal” synthesis parameters are inherently confounded by the choice of growth medium.

As a result, meaningful cross-study comparisons remain highly limited. It is currently not possible to determine whether observed differences in AgNP characteristics arise primarily from intrinsic biological variation between fungal species or from uncontrolled differences in medium composition, including nutrient availability, carbon and nitrogen sources, and trace element profiles.

### 6.4. Temperature

Temperature exerts a complex and often non-linear influence on fungal-mediated AgNP biosynthesis, reflecting the balance between thermal activation of biochemical reduction processes and thermal denaturation of biomolecules.

Most fungal systems exhibit an optimal temperature range between 25 and 50 °C. For example, *Aspergillus sydowii* demonstrated a bell-shaped response, with absorbance values of ~0.35 at 20 °C, ~0.53 at 30 °C, ~0.63 at 40 °C, and a peak of ~0.82 at 50 °C, followed by a decline to ~0.73 at 60 °C [85]. Similarly, *Aspergillus terreus* ITCC 9932.15 reached maximum absorbance (~2.6) at 35 °C, compared to ~1.0 at both 25 °C and 45 °C. Inhibition of nanoparticle formation was observed at temperatures ≥45 °C, with minimal synthesis at 55 °C (~0.3), likely due to enzyme denaturation and loss of reductase activity [96].

Interestingly, several studies have demonstrated efficient AgNP synthesis at elevated temperatures (60–100 °C), indicating the contribution of non-enzymatic reduction pathways. For multiple fungal species, temperatures around 90 °C under alkaline conditions (pH 9–12) produced the smallest and most monodisperse nanoparticles within as little as 1 h. For instance, *Chaetomium thermophilum* produced spherical AgNPs (8.93 ± 2.29 nm) at 90 °C, where enzyme denaturation suggests a predominantly metabolite-driven reduction mechanism [111]. In *Pleurotus floridanus*, high temperatures (80–100 °C) significantly accelerated synthesis, with strong SPR peaks appearing within 30–120 min (absorbance ~2.6–4.0), compared to lower absorbance values at 30–60 °C under the same incubation times [112].

Minimum temperature requirements vary substantially between species. *Trichoderma longibrachiatum* exhibited a sharp optimum at 28 °C, with no detectable nanoparticle formation at either 23 °C or 33 °C [109]. Among marine fungi, *Penicillium citrinum* IBCLP11 required temperatures ≥30 °C, while *Aspergillus niger* IBCLP20 required ≥25 °C; for both strains, temperatures above 35 °C impaired synthesis [108]. At low-temperature extremes, nanoparticle formation was negligible at 4 °C for *Trichoderma reesei* [113] and *Penicillium polonicum* PG21 [91].

Temperature influences multiple aspects of the biosynthesis process, including enzyme activity profiles, biomolecule stability, and the balance between nucleation and growth. Within optimal ranges, elevated temperatures increase nucleation rates and limit secondary reduction on existing nuclei, resulting in smaller nanoparticles [19]. For example, in *Trichoderma reesei*, increasing the temperature to 40 °C resulted in an approximately twofold increase in yield compared to 30 °C after 144 h (61.1 vs. 32.5 mg/L), likely due to enhanced enzyme-mediated reduction and increased formation of Ag^0^ nuclei [21].

Overall, temperature plays a dual and highly system-dependent role in fungal AgNP biosynthesis. While moderate temperatures favor enzyme-mediated reduction, efficient synthesis at elevated temperatures highlights the significant contribution of non-enzymatic pathways, which are often underrepresented in the literature. The wide variability in optimal and threshold temperatures across fungal species further indicates that temperature cannot be generalized as a universal parameter. Future studies should therefore aim to distinguish more clearly between enzymatic and metabolite-driven mechanisms and systematically evaluate their relative contributions under different thermal conditions.

#### Critical Considerations

A major limitation in the current literature is the absence of systematic and controlled investigations into the interplay between temperature and other critical synthesis parameters. Most studies evaluate temperature in isolation, without considering its interaction with pH, precursor concentration, or biomolecule availability, despite clear evidence that these variables are strongly interdependent. As a result, reported “optimal” temperature ranges are highly context-dependent and cannot be reliably generalized across fungal systems.

Another significant gap is the lack of mechanistic differentiation between enzyme-mediated and non-enzymatic reduction processes at varying temperatures. While numerous studies report efficient AgNP synthesis at elevated temperatures (60–100 °C), these conditions likely involve enzyme denaturation and a shift toward metabolite-driven reduction pathways. However, most studies do not explicitly verify this transition or quantify the relative contributions of enzymatic versus non-enzymatic mechanisms. Without targeted experimental approaches—such as enzyme inhibition, proteomic analysis, or fractionation of extracellular components—the underlying reduction pathways remain largely speculative.

Furthermore, temperature-dependent effects on nanoparticle properties are typically assessed using limited endpoints, most commonly UV–Vis absorbance or particle size. Systematic evaluation of how temperature influences colloidal stability, protein corona composition, surface charge, and functional activity is largely lacking. This is particularly important given that elevated temperatures may alter the integrity and composition of capping biomolecules, thereby affecting nanoparticle stability and performance over time.

Additionally, most studies focus on short-term synthesis outcomes, without examining long-term stability or post-synthesis evolution of nanoparticles formed at different temperatures. Processes such as Ostwald ripening, aggregation, and surface restructuring may be strongly influenced by synthesis temperature, yet they remain underexplored. Similarly, no studies have evaluated the reproducibility or scalability of temperature-optimized protocols, especially under industrially relevant conditions.

Finally, despite the observed variability in optimal temperatures across fungal species, comparative studies under standardized conditions are scarce. Without systematic, head-to-head comparisons, it is not possible to distinguish species-specific thermal responses from methodological variability. Addressing these limitations will be critical for developing predictive frameworks that link temperature-dependent biochemical processes with nanoparticle formation mechanisms and properties.

### 6.5. pH

pH is one of the most influential parameters in fungal-mediated AgNP biosynthesis, affecting both enzyme activity and electrostatic stabilization mechanisms.

Most studies identify optimal pH values in the neutral-to-alkaline range (pH 6–10), although the exact optimum varies among fungal systems. For example, *Aspergillus terreus* ITCC 9932.15 exhibited maximal nanoparticle formation at pH 8, with peak absorbance reaching ~2.0, compared to ~1.3–1.4 at pH 6, ~0.25 at pH 4, and ~0.15–0.2 at pH 10–12 [96]. Similarly, *Trichoderma harzianum* MTCC 795 showed optimal formation at pH 10 for rapid synthesis; however, increasing pH from 8 to 11 increased SPR intensity but also caused a red shift from ~420 nm to ~460 nm, indicating the formation of larger nanoparticles [100].

Acidic pH conditions frequently suppress or completely inhibit nanoparticle formation. In *Aspergillus oryzae* MTCC 1846, no synthesis was observed at pH 4–5, with nanoparticle formation initiating at pH ≥6 and reaching a maximum at pH 10. Under optimal conditions, the reaction was completed in approximately 30 min, whereas no detectable formation occurred at lower pH values [19]. Similarly, *Phellinus adamantinus* showed no AgNP formation at pH 3–5, while neutral pH (pH 7) resulted in stable nanoparticles with a sharp SPR peak at 418 nm [90].

Alkaline conditions are frequently associated with enhanced nanoparticle yield. For *Fusarium oxysporum*, alkaline pH was essential for effective synthesis. In *Pleurotus floridanus*, pH 11 produced the highest and most well-defined SPR peaks, whereas little or no nanoparticle formation occurred under acidic to near-neutral conditions (pH 3–7) [112]. A near-linear increase in absorbance (R^2^ ≈ 0.96) was observed in *Neopestalotiopsis clavispora* across a broad pH range, from ~0.036 at pH 2 to ~0.124 at pH 12 [104]. However, excessively high pH values may compromise nanoparticle stability. For instance, in *Talaromyces funiculosus*, pH 10.5 led to protein inactivation, resulting in aggregation and broadened SPR bands [89].

**Figure 7:**
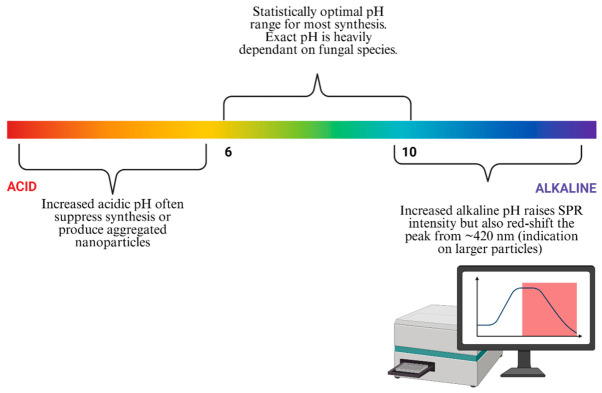
Generalised diagram of optimal pH range for extracellular AgNP biosynthesis. Created in BioRender. Vorkapić, M. (2026) https://BioRender.com/5y4859o (accessed on 8 June 2026).

The effects of pH can be explained through multiple mechanistic pathways (Figure 7). Alkaline pH enhances the reduction process by increasing the availability of OH^−^ ions, which can participate in electron transfer and promote the ionization of functional groups in biomolecules involved in reduction [114]. In addition, higher pH values facilitate adsorption of OH^−^ onto nanoparticle surfaces, stabilizing capping proteins and reducing aggregation [105]. In contrast, acidic conditions may lead to protein denaturation and reduced enzymatic activity [88]. Moreover, the use of strong acids such as HCl for pH adjustment may result in AgCl formation, thereby inhibiting nucleation [115].

Importantly, the pH that maximizes nanoparticle formation does not always coincide with the pH that ensures optimal stability. For example, *Aspergillus templicola* OR480102 exhibited maximal formation at pH 11, but the resulting nanoparticles were neutralized post-synthesis to ensure stability during bioassays [116]. Similarly, marine fungal systems demonstrate optimal stability under mildly acidic to neutral conditions, as observed for *Penicillium citrinum* IBCLP11 (pH 6.5) and *Aspergillus niger* IBCLP20 (pH 6.0); outside the pH range of 6.0–7.5, precipitation occurred within one week [117].

The biomolecular templates secreted by fungi (proteins, organic acids, and polysaccharides) carry ionizable functional groups, most importantly carboxyl (-COOH) and hydroxyl (-OH) moieties, whose protonation state is dictated by the ambient pH. Below their characteristic pKa values these groups are protonated and electrically neutral, presenting no preferential affinity for ionic species in solution. As pH rises above those pKa values, deprotonation generates carboxylate (-COO^−^) and phenolate (-O^−^) anions, endowing the molecular template with a net negative surface charge. Because Ag^+^ is a monovalent cation, this charge inversion creates a direct Coulombic attraction between the template and the silver ion. The electrostatic potential energy of the Ag^+^-COO^−^ interaction is negative (attractive), drawing silver ions toward the template surface and increasing their local concentration around the electron-donating functional groups well before any reduction event takes place. This pre-concentration effect is not trivial, carboxylate oxygen has been confirmed as the primary anchoring site for Ag^+^ in model capping systems [118], and molecular dynamics simulations of carboxylate-bearing polymers show that increasing the degree of ionization (the computational analogue of raising pH) progressively strengthens polymer-to-AgNP interactions through exactly this electrostatic mechanism, while fully protonated, neutral chains bind far more weakly [119]. The Coulombic attraction therefore serves a dual purpose: it concentrates Ag^+^ in the immediate coordination environment of the reducing groups, shortening the electron-transfer distance and favouring the inner-sphere pathway, and it anchors the newly formed Ag^0^ nuclei to the template surface, where continued capping by the deprotonated ligands stabilises the growing nanoparticle against aggregation. The net result is that alkaline conditions simultaneously maximise the electrostatic driving force for Ag^+^ adsorption and the stabilising capacity of the molecular corona (both consequences of the same deprotonation chemistry).

Collectively, these findings highlight that pH governs multiple aspects of AgNP biosynthesis, including enzyme activity, reduction kinetics, and nanoparticle stability. While alkaline conditions generally promote efficient reduction, excessive pH values may disrupt biomolecular stabilization mechanisms, underscoring the need for system-specific optimization of pH conditions.

#### Critical Considerations

A pervasive limitation across the included studies is the lack of standardized, multi-point pH titration protocols combined with comprehensive nanoparticle characterization at each pH level. Most studies identify “optimal” pH values based solely on UV–Vis absorbance intensity [85,120], which serves only as an indirect proxy for nanoparticle yield and conflates particle concentration with size-dependent extinction effects. Only a limited number of studies provide detailed structural characterization—such as TEM-derived size distributions or DLS-based polydispersity indices—across multiple pH conditions [106,115,121,122]. This reliance on surrogate endpoints significantly limits the ability to establish quantitative relationships between pH, nanoparticle size, and yield.

Furthermore, reported optimal pH values span a remarkably broad range, from as low as 4.5 to as high as 12 [104,106,123], indicating substantial species-dependent variability that has not been systematically investigated. Most studies examine a single fungal isolate under highly specific experimental conditions, including variations in culture medium composition, temperature, AgNO_3_ concentration, and even light exposure [20,104,115]. This introduces significant confounding effects, making it difficult to isolate the independent contribution of pH.

Only a small number of studies have applied factorial or response surface methodology (RSM) designs capable of evaluating interaction effects [124,125]. However, even these did not report interaction coefficients for pH in combination with other key parameters such as temperature or precursor concentration. Consequently, it remains unclear whether the wide variability in optimal pH values reflects genuine biological differences in fungal reductase systems or arises from uncontrolled experimental variables.

Addressing these limitations will be essential for establishing mechanistically meaningful pH–response relationships and for enabling the rational optimization of fungal-mediated AgNP biosynthesis.

### 6.6. Biomass and Extract Composition

Biomass quantity—expressed either as the amount of wet mycelium used for the preparation of cell-free extracts or as the extract volume in the reaction mixture—significantly influences AgNP biosynthesis, typically exhibiting optimal ranges with diminishing returns or inhibitory effects at higher levels.

In *Trichoderma longibrachiatum*, increasing fungal biomass from 1 to 20 g led to a progressive increase in color intensity and UV–Vis absorbance. However, 10 g of biomass produced the most favorable nanoparticles, characterized by small, monodisperse particle sizes (~5–25 nm) and enhanced stability (zeta potential −19.7 mV) [109]. Further increase to 15 g resulted in aggregation and reduced colloidal stability (zeta potential −4.33 mV). Similarly, in *Trichoderma reesei*, increasing the biomass extraction ratio (5%, 10%, and 15% wet biomass in water) led to proportional increases in both yield and biotransformation efficiency. After 168 h at 10 mM AgNO_3_, the degree of biotransformation increased from 3.6% to 6.2% and 7.0%, while AgNP concentrations increased from 39.2 to 67.0 and 75.2 mg/L, respectively [21].

Biomass concentration plays a crucial role in fungal-mediated silver nanoparticle synthesis, as it determines the availability of extracellular enzymes and metabolites responsible for reduction processes. Variations in biomass production among fungal strains directly influence the efficiency of AgNP formation [126]. This effect has been attributed to an excess of reducing biomolecules, which may disrupt the balance between nucleation and growth processes.

Across studies, optimal biomass concentrations are frequently reported in the range of 5–15 g per 100 mL or equivalent ratios. For example, *Talaromyces funiculosus* exhibited optimal nanoparticle morphology and stability at 5 g biomass, whereas higher levels (10–20 g) resulted in broadened SPR bands and visible precipitation [89]. Similarly, *Saccharomyces ellipsoideus* BSU-XR1 showed optimal performance at 10 g among the tested range (5–20 g), producing nanoparticles with an average size of 17.2 nm [92].

When biomass content was held constant and the extract volume in the reaction mixture was varied, similar optimal ranges were observed. In *Hypocrea lixii* GGRK4, culture filtrate supernatant (CFS) volume showed a positive linear effect on nanoparticle formation in one-factor-at-a-time (OFAT) experiments (0.25–2.25 mL range). However, multivariate optimization using response surface methodology (RSM) identified 1.931 mL as the optimal volume when combined with specific silver nitrate concentrations and incubation times [124].

Collectively, these findings indicate that biomass and extract composition are critical determinants of both nanoparticle yield and physicochemical properties. While increasing biomass generally enhances reducing capacity, excessive amounts can disrupt controlled nucleation and stabilization processes, highlighting the importance of balanced optimization.

#### Critical Considerations

A major limitation across the current literature is the lack of systematic investigation into the quantitative relationship between biomass loading, extract composition, and nanoparticle formation kinetics. Most studies evaluate biomass as a single variable using one-factor-at-a-time (OFAT) approaches, without accounting for its interactions with key parameters such as silver precursor concentration, pH, temperature, and extraction conditions. As a result, reported “optimal” biomass levels are highly context-dependent and cannot be generalized across different fungal systems or experimental setups.

Another significant gap lies in the limited characterization of extract composition. While biomass quantity is routinely reported, the biochemical composition of the corresponding extracts—such as protein concentration, enzyme activity, and metabolite profiles—is rarely quantified. Consequently, biomass is often used as a surrogate for reducing capacity, despite the fact that variations in growth conditions, physiological state, and extraction protocols can lead to substantial differences in the composition and activity of the fungal secretome. This lack of molecular-level characterization limits the mechanistic interpretation of observed trends.

Furthermore, the relationship between biomass concentration and nanoparticle properties is typically assessed using a restricted set of endpoints, most commonly UV–Vis absorbance and, less frequently, particle size. Comprehensive evaluation of how biomass influences colloidal stability, capping efficiency, surface charge, and functional performance remains largely absent. In particular, excessive biomass levels may introduce high concentrations of proteins and metabolites that disrupt the balance between nucleation and growth, yet this effect is seldom investigated using detailed structural or surface analyses. In addition, the role of extraction methodology is largely overlooked. Parameters such as extraction time, solvent composition, and biomass-to-solvent ratio can significantly influence the yield and composition of bioactive compounds in the extract, yet they are rarely standardized or systematically optimized. This introduces further variability and complicates cross-study comparisons. Finally, although some studies have explored extract volume as an independent variable, the scaling behavior of biomass-derived systems has not been adequately addressed. No studies have systematically evaluated how biomass concentration impacts reproducibility, batch-to-batch variation, or process scalability. Without such data, it remains difficult to translate laboratory-scale findings into industrially relevant biosynthesis protocols.

Addressing these limitations will be essential for establishing quantitative and mechanistically grounded relationships between biomass loading, extract composition, and AgNP formation, ultimately enabling more predictable and reproducible fungal-mediated biosynthesis systems.

### 6.7. Cross-Parameter Interactions and Limitations in Optimization Studies

A central limitation emerging across all examined parameters—silver precursor concentration, incubation time, culture medium composition, temperature, pH, and biomass/extract composition—is the pervasive reliance on one-factor-at-a-time (OFAT) experimental approaches. While individual studies provide valuable insights into parameter-specific effects, they largely fail to account for the strong interdependence between variables such as pH, temperature, and AgNO_3_ concentration, which collectively govern reduction kinetics and nanoparticle stability [88,93,94,127]. Consequently, reported “optimal” conditions should be interpreted as context-specific rather than universally applicable.

Evidence from individual parameter studies suggests that these interactions are highly significant. For example, the effect of temperature on AgNP formation is strongly modulated by pH, with elevated temperatures favoring non-enzymatic reduction pathways under alkaline conditions [111,112]. Similarly, silver precursor concentration influences not only nucleation rates but also interacts with biomass loading and secretome composition, determining whether reduction leads to controlled nanoparticle formation or aggregation [19,85,126]. Culture medium composition further complicates this landscape by altering enzyme expression and metabolite availability, thereby indirectly modifying the effects of other parameters such as pH and precursor concentration [88,110].

Despite these clear interdependencies, only a limited number of studies have employed factorial or response surface methodology (RSM) approaches capable of capturing interaction effects [124,125,126,127]. Even in these cases, comprehensive interaction modeling remains incomplete, with minimal reporting of interaction coefficients or mechanistic interpretation of multivariate effects. As a result, the current body of literature remains largely fragmented, with limited predictive capacity.

Another critical limitation lies in the lack of standardized optimization criteria across studies. Most investigations rely on UV–Vis absorbance as a primary indicator of nanoparticle formation [85,95,102], often without simultaneous evaluation of size distribution, colloidal stability, or functional performance. As demonstrated across multiple sections, parameters that maximize nanoparticle yield do not necessarily produce particles with optimal size, monodispersity, or bioactivity. This disconnect complicates the identification of truly optimal synthesis conditions for practical applications.

Furthermore, variability in experimental design—particularly with respect to culture media, biomass preparation, and extraction protocols—introduces additional layers of confounding [88,104,109]. Since these factors directly influence the composition of the fungal secretome, they also modulate the identity and activity of biomolecular reducing and capping agents. This variability makes it difficult to distinguish whether observed differences in nanoparticle characteristics arise from intrinsic biological differences between fungal species or from methodological inconsistencies.

Collectively, these limitations highlight the need for a shift from empirical, single-parameter optimization toward integrative, multivariate approaches that account for cross-parameter interactions. The application of statistically designed experiments, combined with detailed biochemical and physicochemical characterization, will be essential for developing predictive models of fungal-mediated AgNP biosynthesis. Such approaches are critical for advancing the field toward reproducible, scalable, and mechanistically grounded synthesis strategies. Comparative data illustrating the effects of synthesis parameters on AgNP size, yield, and stability across fungal systems are summarized in Table 2.

## 7. Characterization of Fungal-Derived AgNPs

**Figure 8:**
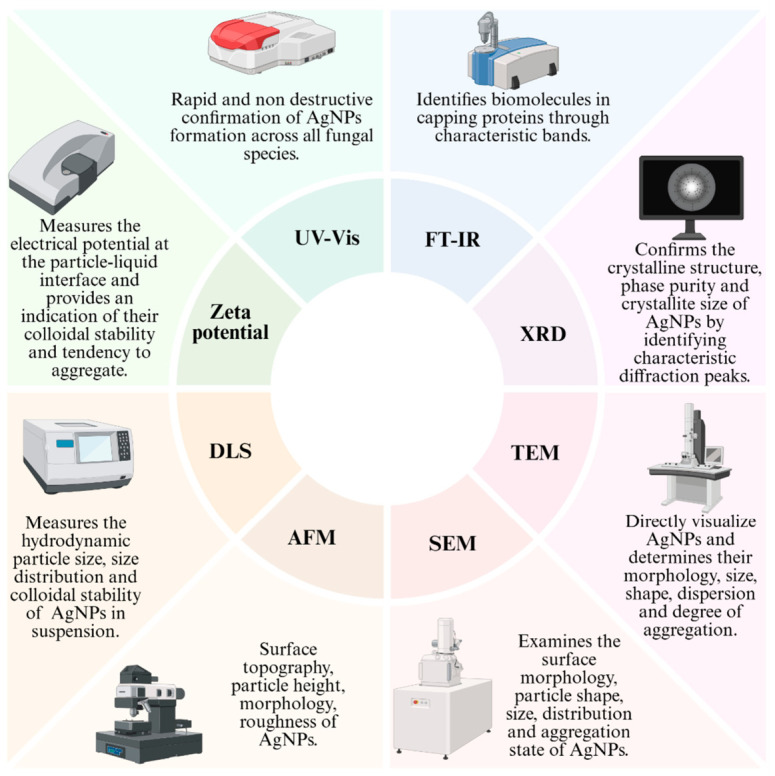
Schematic overview of instrumental methods of analysis. Created in BioRender. Vorkapić, M. (2026) https://BioRender.com/h5nhqxz (accessed on 8 June 2026).

The characterization of fungal-mediated AgNPs relies on a combination of complementary analytical techniques, each providing specific insights into their optical, structural, morphological, and colloidal properties (Figure 8). Given the complex nature of biogenic nanoparticles, which are typically coated with a heterogeneous layer of biomolecules derived from fungal secretomes, no single method is sufficient to fully describe their physicochemical characteristics. Structural and morphological characterization is particularly important for understanding key nanoparticle features, including size, shape, crystallinity, and surface architecture, which directly influence their stability, reactivity, and potential applications. Techniques such as X-ray diffraction (XRD), transmission electron microscopy (TEM), and scanning electron microscopy (SEM) provide complementary insights into these properties. A summary of commonly employed analytical techniques and their roles in AgNP characterization is presented in Table 1.

Optical techniques, particularly UV–Vis spectroscopy, are widely used for the initial confirmation of nanoparticle formation through the detection of surface plasmon resonance (SPR) bands [128,129]. Spectroscopic methods such as Fourier-transform infrared (FTIR) spectroscopy enable the identification of functional groups involved in reduction and stabilization processes, providing insight into nanoparticle–biomolecule interactions [35,130]. Structural and crystallographic properties are most commonly evaluated using X-ray diffraction (XRD), which confirms phase composition and crystalline structure [131,132].

Electron microscopy techniques, including transmission electron microscopy (TEM) and scanning electron microscopy (SEM), provide detailed information on nanoparticle morphology, size, and aggregation state, while also allowing visualization of capping layers and particle distribution [109,133]. In parallel, dynamic light scattering (DLS) and zeta potential measurements are essential for evaluating hydrodynamic size and colloidal stability in aqueous environments, reflecting nanoparticle behavior under biologically relevant conditions [109,113].

Importantly, discrepancies between analytical techniques are frequently observed due to differences in measurement principles, particularly when comparing dry-state imaging methods (e.g., TEM, SEM) with solution-based techniques (e.g., DLS) [35,109]. Therefore, a multi-technique approach is essential to obtain a comprehensive understanding of fungal-derived AgNPs and to ensure accurate interpretation of their physicochemical properties and functional performance.

### 7.1. Optical and Spectroscopic Techniques

#### 7.1.1. Ultraviolet–Visible Spectroscopy (UV-VIS)

UV–Vis spectroscopy is the most widely used technique for the initial confirmation of AgNP formation, as it reliably detects surface plasmon resonance (SPR) bands characteristic of silver nanoparticles.

This technique is highly effective for rapid and non-destructive monitoring of nanoparticle synthesis across a wide range of fungal systems. Characteristic SPR bands are typically observed in the range of 380–456 nm [128]. In addition to confirming nanoparticle formation, UV–Vis spectroscopy is frequently used to monitor synthesis kinetics [109], assess colloidal stability over time [129], and optimize synthesis parameters such as pH, temperature [130], and AgNO_3_ concentration [30]. The position of the SPR peak provides qualitative insights into particle size, where blue shifts indicate smaller nanoparticles [123], while red shifts suggest larger particles or aggregation phenomena [128].

Several studies have also reported additional absorption bands in the 265–280 nm region, which are commonly attributed to aromatic amino acids (e.g., tryptophan, tyrosine, and phenylalanine) present in fungal proteins [131]. These signals provide indirect evidence of protein-based capping and nanoparticle–biomolecule interactions.

Sample preparation for UV–Vis analysis is generally minimal, typically requiring direct measurement of the colloidal reaction mixture [132]. In some cases, dilution is applied to ensure measurements fall within the linear detection range [109], while baseline correction may be performed using the original fungal filtrate [34]. Spectral scans are commonly recorded over the 200–800 nm range, with resolutions of 0.5–1 nm where reported [133]. Despite its widespread use, UV–Vis has several limitations. The technique does not provide quantitative information on particle size distribution or accurately resolve polydispersity beyond qualitative assessment based on peak broadening [115]. It also lacks morphological information [134], and discrepancies in reported SPR peak positions can arise due to variations in experimental conditions [135]. Importantly, UV–Vis spectroscopy cannot distinguish between core nanoparticle size and hydrodynamic diameter [34], necessitating the use of complementary characterization techniques such as electron microscopy and dynamic light scattering.

#### 7.1.2. Fourier-Transform Infrared (FTIR) Spectroscopy 

FTIR spectroscopy is widely used to identify functional groups involved in nanoparticle reduction and stabilization, providing key insights into nanoparticle–biomolecule interactions. In fungal-mediated AgNP synthesis, FTIR analysis consistently indicates the presence of proteins as primary capping agents.

Characteristic amide I bands (C=O stretching) are typically observed in the range of 1620–1650 cm^−1^, while amide II bands (N–H bending and C–N stretching) appear at 1516–1557 cm^−1^, confirming the involvement of proteinaceous components. Hydroxyl groups associated with phenolic compounds and alcohols are commonly detected in the range of 3200–3600 cm^−1^ [128], while carboxylate (COO^−^) groups appear between 1380 and 1590 cm^−1^ [131].

Sample preparation methods vary considerably depending on the experimental setup. The most commonly used approach involves preparing KBr pellets by mixing dried AgNP powder with potassium bromide (typically at ratios of 1:100 or 2:200 mg) and pressing the mixture into semi-transparent disks for transmission measurements [21]. Alternatively, attenuated total reflectance (ATR) mode enables direct analysis of dried nanoparticle samples without the need for pellet preparation [40]. Other approaches include diffuse reflectance FTIR spectroscopy (DRIFTS) [136], drop-casting followed by air-drying on glass substrates [137], and direct measurements of aqueous suspensions in quartz cuvettes [138].

FTIR analysis is particularly effective for detecting interactions between biomolecules and nanoparticle surfaces, as evidenced by shifts in characteristic functional group frequencies. For example, Li et al. observed a shift in the amide I band from 1640 to 1627 cm^−1^, attributed to electrostatic interactions between AgNP surfaces and protein amide groups [35]. Mahmoud et al. reported similar shifts (1650 to 1627 cm^−1^) in Ag–chitosan systems, indicating silver binding to chitosan functional groups [139]. Constantin et al. observed band broadening and shifts from 1635.63 to 1647.85 cm^−1^, suggesting oxidation of carbonyl groups and their participation in silver ion reduction [129]. In addition, Pavić et al. identified a characteristic Ag–O band at ~521 cm^−1^, detected only in AgNP samples and absent in the original extract, providing further evidence of metal–oxygen interactions [140]. Despite its utility, FTIR has several limitations. The technique provides information primarily at the level of functional groups and cannot unambiguously identify specific capping molecules [136]. Broad and overlapping absorption bands, particularly in the O–H/N–H region, can complicate spectral interpretation. For instance, Ballottin et al. reported that overlapping protein bands in KBr pellets required complementary Raman spectroscopy to distinguish between S–Ag and N–Ag interactions [34]. Similarly, Fernández et al. observed a broad and poorly resolved O–H/N–H region in *Cryptococcus laurentii*, necessitating spectral deconvolution to resolve individual amide contributions [141].

Overall, FTIR represents a powerful tool for probing nanoparticle surface chemistry, particularly in biogenic systems, where complex mixtures of proteins and metabolites mediate both reduction and stabilization processes.

### 7.2. Structural and Morphological Analysis

#### 7.2.1. X-Ray Diffraction (XRD)

X-ray diffraction (XRD) is widely used to determine the crystalline structure and phase composition of fungal-derived AgNPs. The characteristic face-centered cubic (fcc) silver reflections—(111), (200), (220), and (311) are consistently observed at 2*θ* values of approximately 38°, 44°, 64–65°, and 77–78°, respectively [117]. Among these, the (111) reflection is typically the most intense, indicating preferential crystallographic orientation along this plane [129]. In some cases, an additional (222) reflection is detected at approximately 81–82° 2*θ* [136].

Sample preparation for XRD typically involves isolating AgNPs by centrifugation, followed by washing and drying to obtain powders suitable for powder diffraction analysis [130]. Alternative approaches include drop-casting AgNP suspensions onto substrates such as glass slides to form thin films [133], or analyzing semi-solid or gel-like samples without extensive drying [129]. Measurements are generally performed using Cu Kα radiation (λ = 1.5406–1.5418 Å), with operating conditions in the range of 40–50 kV and 30–40 mA [133]. Crystallite size estimation is commonly performed using the Scherrer equation; however, only a small proportion of studies report such calculations. Among the available data, crystallite sizes range from approximately 11 to 121 nm. For instance, Owaid et al. reported a mean crystallite size of 43.9 nm for *Agaricus bisporus*-derived AgNPs, with individual peak-based estimates ranging from 15.4 to 41.5 nm [142]. Pavić et al. estimated crystallite sizes of ~24 nm for *Fomes fomentarius* AgNPs [140], while Baker et al. reported ~11 nm for *Alternaria*-mediated nanoparticles [143]. In contrast, Dandapat et al. obtained larger crystallite sizes ranging from 60.6 to 121.2 nm (average ~102.1 nm) for *Ganoderma applanatum* [135], and Saqib et al. reported ~39 nm for *Aspergillus fumigatus*-derived AgNPs [133].

In addition to the characteristic fcc reflections, several studies have reported minor or secondary peaks. Ninganagouda et al. identified a peak at approximately 32° 2*θ*, attributed to AgCl formation [132]. Similarly, Pereira et al. observed additional peaks at 32.08°, 46.00°, 54.6°, and 57.3°, which were assigned to possible AgCl or Ag_2_O phases arising from precursor or medium-derived contaminants [144]. Other studies reported unidentified peaks attributed to organic biomolecules associated with the fungal extract. For example, Saqib et al. described additional peaks marked by asterisks, attributed to fungal metabolites [133], while Dandapat et al. associated unassigned peaks with bio-organic capping materials acting as stabilizing agents [135]. Comparable observations were reported by Lotfy et al., who linked minor peaks to residual reducing and capping biomolecules [145]. Despite its widespread use, XRD analysis of fungal-derived AgNPs presents several limitations. The presence of organic capping layers often results in broad amorphous backgrounds, complicating peak identification and analysis [129]. In addition, peak broadening associated with nanocrystalline domains limits detailed structural refinement and may reduce accuracy in crystallite size estimation [130]. Many studies provide only qualitative phase identification without reporting lattice parameters or quantitative crystallinity indices [128]. Notably, a substantial proportion of studies (12 out of 41) did not employ XRD analysis, instead relying on alternative techniques such as high-resolution TEM combined with selected area electron diffraction (SAED) to confirm crystallinity and phase composition. While these approaches provide valuable structural information, the absence of XRD data limits cross-study comparability and standardization. These findings highlight the importance of combining XRD with complementary techniques to accurately interpret crystallinity and distinguish between metallic phases and biomolecule-associated signals in biogenic systems.

#### 7.2.2. Transmission Electron Microscopy (TEM)

Transmission electron microscopy (TEM) is the most widely used imaging technique for characterizing fungal-derived AgNPs, appearing in the majority of reported studies. TEM provides direct visualization of nanoparticle morphology, size, and aggregation state at the nanoscale.

Across fungal systems, TEM analysis consistently reveals predominantly spherical to quasi-spherical nanoparticle morphologies, with particle sizes ranging from small clusters of 1–4 nm [21] to larger particles exceeding 60 nm [139]. High-resolution TEM (HRTEM), employed in a subset of studies, enables visualization of atomic lattice fringes and provides direct evidence of crystallinity. Measured interplanar spacings (d-spacings) typically fall within the range of 0.23–0.267 nm, corresponding to the (111) lattice planes of face-centered cubic (fcc) silver [21]. Selected area electron diffraction (SAED) patterns further confirm crystallographic structure, showing concentric rings or discrete spots indexed to the (111), (200), (220), and (311) planes [40]. These results complement or, in some cases, substitute for XRD-based structural analysis.

Sample preparation for TEM is relatively straightforward and consistent across studies. The standard procedure involves depositing a small aliquot (typically 5–10 µL) of AgNP colloidal suspension onto carbon-coated copper grids [110], followed by drying under ambient conditions [40] or in a vacuum desiccator [109]. Variations in drying protocols have been reported, including infrared light exposure for accelerated drying [142] and short-term irradiation using mercury lamps [146]. Various grid types are used, including Formvar-coated [40], Butvar B-98-coated [140], and lacey carbon grids for high-resolution imaging [128], typically supported by 200–300 mesh copper grids.

A critical consideration when interpreting TEM data is the discrepancy between particle sizes obtained by different characterization techniques. TEM-derived measurements consistently yield smaller nanoparticle sizes compared to hydrodynamic sizing methods such as dynamic light scattering (DLS). For example, Gemishev et al. reported mean TEM diameters of 8.2 and 5.1 nm, while corresponding DLS Z-average values were 190.8 and 116.8 nm, respectively, reflecting the contribution of hydrated capping layers not visible in dried TEM samples [113]. Similarly, Ballottin et al. reported core particle sizes of 35 ± 10 nm by TEM compared to hydrodynamic diameters of 264.9 ± 3.2 nm measured by DLS [34], while Elamawi et al. observed TEM sizes of approximately 10 nm versus DLS values of 24.43–29.15 nm [109].

These discrepancies highlight the importance of distinguishing between core nanoparticle size and hydrodynamic behavior in solution. While TEM provides high-resolution structural information, it cannot capture the dynamic physicochemical environment of nanoparticles in colloidal systems. Therefore, TEM must be complemented with solution-based techniques such as DLS to obtain a comprehensive understanding of nanoparticle properties. Such differences are particularly pronounced in biogenic systems due to the presence of complex protein and metabolite capping layers.

#### 7.2.3. Scanning Electron Microscopy (SEM)

Scanning electron microscopy (SEM) is widely used to examine nanoparticle surface morphology, aggregation behavior, and overall topography. Compared to TEM, SEM typically reports larger apparent particle sizes, primarily due to aggregation during sample drying and the presence of biomolecular surface coatings [139].

Several studies have highlighted this effect. For example, Konappa et al. reported an average particle size of 72 nm by SEM, while DLS measurements for the same system yielded a significantly smaller value of 21.49 nm [31], demonstrating the influence of drying-induced aggregation. Similarly, Ahmed et al. noted that SEM images revealed “aggregated particles due to the capping agent(s), mainly metabolites,” resulting in larger apparent sizes compared to TEM and XRD measurements [147].

Sample preparation for SEM generally involves depositing AgNP suspensions as thin films onto suitable substrates, followed by drying [134]. To improve imaging quality and prevent charging, conductive coatings are often applied. Reported coatings include thin gold layers (~5 nm) [129], gold–palladium alloys [133], and platinum coatings [136]. However, some studies have successfully imaged AgNPs without additional coating [111], likely due to the intrinsic conductivity of metallic silver. Common substrates include glass slides [47], carbon-coated copper grids [146], and standard SEM stubs [126]. When SEM and TEM are used in parallel, TEM consistently provides more accurate determination of primary particle size. For instance, Saqib et al. reported SEM-derived sizes of 65–85 nm for *Aspergillus fumigatus*-derived AgNPs, whereas TEM analysis revealed smaller particles in the range of 20–60 nm, with an average size of 30 ± 6.05 nm [133]. Similarly, Barkhade et al. observed primary particle sizes of 9–15 nm by TEM for *Penicillium* species, while SEM images showed aggregated structures with apparent sizes of 25.32–31.87 nm [134]. These differences reflect the complementary nature of the two techniques. While TEM provides high-resolution imaging of individual nanoparticles, SEM is particularly valuable for assessing surface topology and aggregate morphology. When combined with energy-dispersive X-ray spectroscopy (EDS), SEM also enables elemental analysis. SEM–EDS analysis confirms the presence of elemental silver through characteristic Ag peaks at approximately 3 keV. For example, Mahmoud et al. reported approximately 80 wt% Ag in both AgNPs and Ag–chitosan nanocomposites [139], while Lotfy et al. measured 68.6 wt% Ag along with minor contributions from Al, P, S, Cu (from the grid), and Zn [135]. In addition, SEM–EDS frequently detects elements associated with fungal capping layers, including C, O, Na, and Cl [133], thereby supporting FTIR-based evidence of biomolecule-mediated stabilization.

Despite its utility, SEM has inherent limitations in resolving primary nanoparticle size, particularly in systems prone to aggregation. Therefore, SEM is most effectively used in combination with complementary techniques such as TEM, XRD, and DLS to obtain a comprehensive characterization of fungal-derived AgNPs. Such discrepancies are particularly pronounced in biogenic systems due to the presence of complex organic capping layers that influence particle agglomeration during sample preparation.

#### 7.2.4. Atomic Force Microscopy (AFM)

Atomic force microscopy (AFM) is a high-resolution surface characterization technique that provides three-dimensional topographical information at the nanoscale. Although less commonly used than electron microscopy methods, AFM offers complementary insights into surface morphology, particle height, and nanoscale roughness of fungal-derived AgNPs.

AFM was employed in only a small number of studies (3 out of 41) but yielded detailed information on nanoparticle surface structure. For instance, Ninganagouda et al. obtained two-dimensional height images (0–0.5 µm scale) and three-dimensional surface profiles over scanning areas of 20 µm × 20 µm, revealing peaks and valleys with heights up to approximately 0.97 µm, consistent with smooth and predominantly spherical nanoparticles [132]. Owaid et al. utilized AFM to assess surface roughness changes induced by ultraviolet (UV) irradiation, reporting an increase in average roughness from 15.4 nm to 33.6 nm after 1 h of exposure at 256 nm. In addition, particle size distributions derived from AFM/SPM data showed a decrease in average diameter from 103.57 nm to 69.47 nm, accompanied by a reduction in particle aggregation (cumulation decreasing from 74.79% to 45.03%) [142]. Chowdhury et al. performed AFM analysis in tapping mode using silicon cantilevers (approximately 135 µm length, ~8 nm tip diameter, spring constant 20–80 N/m, and resonance frequency 276–318 kHz). The sample preparation involved depositing 10 µL of AgNP suspension onto freshly cleaved muscovite mica, allowing adsorption for 15–30 min, followed by vacuum drying, rinsing with Milli-Q water, and subsequent drying. AFM height profiles indicated that most nanoparticles exhibited heights below 50 nm [47]. Similarly, Ahmed et al. obtained three-dimensional topographical maps over approximately 12.4 µm × 12.4 µm scan areas, revealing nanoscale height variations between −35 nm and +50 nm, with an average particle size of approximately 21.5 nm based on histogram analysis [147].

A key advantage of AFM is the ability to analyze immobilized nanoparticles, which minimizes aggregation artifacts commonly observed in SEM imaging. As noted by Ahmed et al., aggregation “cannot occur” under AFM measurement conditions, allowing more accurate assessment of individual particle topography [147].

Despite these advantages, the limited application of AFM across studies suggests that it is not considered essential for routine characterization of fungal-mediated AgNPs. This is likely due to the widespread availability and higher throughput of TEM for morphological analysis and DLS for size measurements in solution. In addition, AFM requires more specialized sample preparation and longer analysis times, which may limit its practical use in large-scale studies.

Overall, AFM serves as a valuable complementary technique, particularly for investigating surface roughness, particle height distribution, and nanoscale topography, where conventional electron microscopy methods may provide limited information.

### 7.3. Colloidal Stability and Particle Size Analysis

#### Dynamic Light Scattering (DLS) and Zeta Potential (ζ)

Dynamic light scattering (DLS) is widely used to determine the hydrodynamic size of nanoparticles in their native aqueous state. In fungal-mediated AgNP systems, DLS measurements consistently report larger particle sizes compared to TEM-derived core diameters, reflecting the contribution of solvation layers and adsorbed biomolecular coronas. This systematic discrepancy arises from fundamental differences between the techniques: DLS measures the effective hydrodynamic diameter of nanoparticles in solution, while TEM visualizes dried particle cores [109]. For example, Gemishev et al. reported TEM diameters of 8.2 and 5.1 nm, compared to DLS Z-average values of 190.8 and 116.8 nm, respectively [113]. Similarly, Li et al. observed TEM sizes of 3.8 ± 1.1 nm and 9.1 ± 2.9 nm, while DLS measurements yielded significantly larger values of 46.30 and 60.09 nm, highlighting the tendency of DLS to overestimate particle size [35].

Polydispersity index (PDI) values provide additional insight into particle size distribution. Reported PDI values range from 0.074 (indicating near-monodisperse systems) [138] to 0.549 (indicating significant heterogeneity) [35]. Lower PDI values are generally associated with improved colloidal stability and uniform nanoparticle populations [109]. Sample preparation for DLS is typically minimal, with measurements performed directly on colloidal suspensions [131]. However, preparatory steps such as dilution, filtration (e.g., through 0.22 µm membranes), and ultrasonication are sometimes applied to minimize aggregation and improve measurement accuracy [109]. For instance, Elamawi et al. demonstrated that filtration significantly improved DLS results, transforming broad single peaks into more resolved multimodal distributions [109]. Zeta potential (ζ) provides a quantitative measure of surface charge and is widely used as an indicator of colloidal stability. In general, absolute values greater than approximately 25–30 mV (positive or negative) indicate sufficient electrostatic repulsion to maintain dispersion stability. Most fungal-derived AgNPs exhibit negative zeta potentials, reflecting the adsorption of negatively charged biomolecules such as proteins and carboxylate-containing metabolites.

Strong negative values have been reported for several systems, including *Duddingtonia flagrans* (−49 to −61.9 mV) [40], *Trichoderma reesei* (−45.2 mV) [21], and *Aspergillus versicolor* (−38.2 mV) [148], indicating effective electrostatic stabilization. These values are typically attributed to negatively charged functional groups on capping biomolecules [109].

However, several systems exhibit lower-magnitude zeta potentials, such as *Purpureocillium lilacinum* (−13.0 mV) [138], *Ganoderma applanatum* (−13.8 mV) [135], and *Aspergillus terreus* (−17.5 mV), which would suggest limited electrostatic stabilization. Notably, some nanoparticles with low or even positive zeta potentials remain stable over extended periods. For example, Ballottin et al. reported a positive value of +8.48 ± 0.45 mV for *Aspergillus tubingensis*-derived AgNPs, yet the particles remained stable for at least six months [34]. This behavior was attributed to steric stabilization mediated by protein capping layers.

These observations demonstrate that electrostatic repulsion alone does not fully explain colloidal stability in fungal-derived AgNP systems. Instead, stability often arises from a combination of electrostatic and steric effects associated with biomolecular coatings. Consequently, zeta potential should be interpreted alongside complementary characterization data.

Zeta potential measurements are typically performed on diluted nanoparticle suspensions under controlled conditions, often at neutral pH and in defined ionic environments (e.g., Milli-Q water or dilute electrolyte solutions) [34,83,109,148,149,150]. Variations in sample preparation—including dilution, electrolyte composition, and filtration—can significantly influence measured values and should be carefully standardized.

Overall, DLS and zeta potential measurements provide essential insight into nanoparticle behavior in colloidal systems. However, their interpretation requires careful consideration of methodological limitations and must be integrated with complementary techniques to accurately assess nanoparticle size, stability, and surface properties.

### 7.4. Integrated Critical Considerations in AgNP Characterization

A key limitation across the characterization of fungal-derived AgNPs is the absence of standardized, multi-technique analytical frameworks. While individual methods such as UV–Vis spectroscopy, FTIR, XRD, TEM, SEM, AFM, and DLS provide valuable but distinct insights into nanoparticle properties, no single technique is sufficient to fully describe the physicochemical complexity of biogenic nanoparticles. Consequently, reliance on isolated techniques often results in incomplete or potentially misleading interpretations of nanoparticle size, structure, and stability.

A recurring issue is the inconsistency between measurements obtained using different analytical approaches. For example, particle sizes determined by DLS are consistently larger than those obtained by TEM due to the inclusion of solvation layers and adsorbed biomolecular coronas [109,113]. Similarly, SEM often reports larger apparent particle sizes due to drying-induced aggregation and surface coatings [139,147]. These discrepancies highlight fundamental differences between techniques that measure particles in solution (DLS) versus in dry state (TEM, SEM), complicating direct comparison across studies.

Another major limitation is the widespread reliance on indirect or proxy measurements. UV–Vis spectroscopy, although extensively used to confirm nanoparticle formation through SPR bands [128], cannot provide precise information about size distribution, morphology, or crystallinity. Likewise, FTIR analysis identifies functional groups associated with capping agents but does not allow unambiguous identification of specific biomolecules [136]. XRD, while effective for confirming crystalline structure, is often used qualitatively without detailed analysis of lattice parameters or crystallinity indices [128]. Interpretation of colloidal stability also presents challenges. Zeta potential is frequently used as a key indicator of nanoparticle stability; however, several studies demonstrate that nanoparticles with low or even positive zeta potentials can remain stable due to steric stabilization provided by adsorbed biomolecules [34]. This indicates that electrostatic repulsion alone is insufficient to explain stability in fungal-mediated systems, where protein and metabolite coronas play a dominant role.

In addition, sample preparation procedures vary significantly across techniques and studies, introducing further sources of variability. Drying methods for TEM and SEM can induce aggregation, while filtration, dilution, and electrolyte conditions can significantly influence DLS and zeta potential measurements [109]. These methodological inconsistencies limit reproducibility and hinder meaningful cross-study comparisons.

Another important gap is the limited integration of characterization data with biological function. Most studies focus on structural and physicochemical parameters without systematically linking these properties to biological activity, such as antimicrobial efficacy or cytotoxicity. As a result, the functional relevance of observed nanoparticle characteristics remains underexplored.

Overall, the characterization of fungal-derived AgNPs remains fragmented and method-dependent. Future studies should adopt integrated, multi-technique approaches combined with standardized protocols to improve reproducibility, enable meaningful comparisons, and establish robust relationships between nanoparticle structure, surface chemistry, and functional performance.

## 8. Critical Considerations and Knowledge Gaps

Despite the extensive application of analytical techniques in the characterization of fungal-mediated AgNPs, several important limitations and inconsistencies remain evident across the literature. A key issue is the strong reliance on a limited set of methods—most commonly UV–Vis spectroscopy, TEM, and occasionally FTIR—while comprehensive multi-technique approaches are applied less consistently [116,117]. This selective use of characterization techniques often results in incomplete or potentially misleading interpretations of nanoparticle properties.

A major challenge arises from discrepancies between different measurement principles. Particle size estimates obtained using TEM and DLS frequently differ by an order of magnitude, reflecting the distinction between core nanoparticle size and hydrodynamic diameter influenced by biomolecular coronas [34,109,113]. However, many studies report these values without sufficient critical comparison, which can lead to misinterpretation of nanoparticle size and dispersity.

Similarly, while UV–Vis spectroscopy is widely employed as a primary confirmation tool, its interpretative capacity remains inherently limited. Surface plasmon resonance (SPR) peak position and shape provide only indirect and qualitative information on particle size and aggregation state and cannot substitute for direct structural characterization [132,133,134]. Nevertheless, several studies rely heavily on UV–Vis data without complementary high-resolution techniques.

FTIR analysis, although valuable for identifying functional groups involved in reduction and stabilization, is frequently overinterpreted. The assignment of specific biomolecules based solely on broad and overlapping absorption bands remains speculative in the absence of complementary analytical techniques [34,136]. This is particularly evident in studies attributing distinct roles to proteins, polysaccharides, or secondary metabolites based only on FTIR peak positions.

In the case of XRD, the technique reliably confirms crystallinity and phase composition; however, its application is not universal, with a significant proportion of studies omitting it entirely [132]. Even when used, analyses are often limited to qualitative phase identification without detailed crystallographic refinement or consistent determination of crystallite size.

Electron microscopy techniques, particularly TEM, provide the most reliable information on nanoparticle morphology and primary size. However, sample preparation steps involving drying can alter aggregation states, and the lack of standardized preparation protocols introduces variability between studies [109,133]. SEM, while valuable for surface morphology and elemental analysis via EDS, is less suitable for precise nanoparticle size determination due to aggregation artifacts [139].

Finally, colloidal characterization using DLS and zeta potential highlights nanoparticle behavior in solution, yet these measurements remain highly sensitive to sample preparation conditions, including dilution, filtration, and ultrasonication [109]. Moreover, interpreting zeta potential as the sole indicator of stability can be misleading, as steric stabilization by biomolecular capping layers may play an equally important, or dominant, role [34].

Overall, the reviewed literature highlights the need for standardized, multi-technique characterization strategies. The integration of optical, spectroscopic, microscopic, and colloidal methods is essential for achieving a comprehensive and accurate understanding of fungal-derived AgNPs. Future studies should prioritize methodological consistency, critical cross-technique comparison, and cautious data interpretation to improve reproducibility and avoid overgeneralization.

## 9. Conclusions and Future Perspectives

Fungal-mediated biosynthesis of silver nanoparticles represents one of the most promising strategies within green nanotechnology, combining sustainable production with the remarkable biochemical versatility of fungal systems. Current evidence indicates that AgNP formation is not governed by a single dominant mechanism but rather by a complex network of interacting biomolecules, including enzymes, proteins, polysaccharides, and secondary metabolites. These components collectively mediate silver ion reduction, nanoparticle nucleation, growth, and stabilization. Although nitrate reductases, oxidoreductases, and redox-active metabolites have been most frequently implicated, their relative contributions remain unresolved and appear to be strongly species- and condition-dependent. This reinforces the central conclusion of this review: there is no universal biomolecular driver of silver reduction in fungal systems.

Despite substantial progress, mechanistic understanding of fungal-mediated AgNP biosynthesis remains limited. Most studies rely on indirect spectroscopic evidence and crude extracellular extracts, while causal validation of specific biomolecular functions is largely lacking. The limited integration of advanced approaches—such as proteomics, metabolomics, and targeted biochemical assays—continues to constrain the ability to distinguish correlation from causation. In addition, variability in experimental design, insufficient standardization of protocols, and inconsistent reporting practices hinder reproducibility and meaningful cross-study comparison.

Future research should move beyond descriptive synthesis studies toward mechanistically resolved and predictive models of fungal AgNP biosynthesis. Priority should be given to the identification and isolation of key biomolecular drivers, elucidation of their synergistic interactions, and establishment of direct causal relationships between secretome composition and nanoparticle properties. The integration of multi-omics approaches with functional validation strategies including enzyme inhibition, fractionation, and reconstitution experiments will be essential. Moreover, systematic optimization using multivariate experimental designs and standardized reporting frameworks is required to improve reproducibility and scalability.

The multiparametric and nonlinearly coupled nature of AgNP synthesis presents a compelling case for machine learning (ML) as a complementary tool: models trained on systematically compiled datasets can navigate the high-dimensional parameter space that classical one-factor optimisation cannot efficiently explore. ANNs applied to green biosynthesis data achieve size predictions with R^2^ above 0.98, and Shapley-value analysis of the literature-derived AgNP datasets identifies synthesis duration, scale, and capping agent choice as the dominant determinants of particle size. Coupled with microfluidic platforms, ML has enabled self-driving synthesis systems capable of autonomously converging on targeted particle properties. Nevertheless, the utility of these approaches remains constrained by the same factors that limit mechanistic understanding of biosynthesis: datasets compiled from heterogeneous experimental protocols transfer poorly across systems, and high predictive accuracy does not resolve the underlying chemistry. Machine learning can therefore be viewed as a powerful empirical complement to mechanistic research but not as a substitute for it (the two approaches will ultimately need to converge for truly controlled and reproducible biogenic AgNP synthesis to be realised).

Addressing these challenges will not only advance fundamental understanding of fungal nanobiotechnology but also enable the rational design and industrial translation of biologically synthesized silver nanoparticles. Such progress is critical for unlocking their full potential in biomedicine, environmental applications, and advanced material systems.

## Figures and Tables

**Table 1:** Key studies on fungal-mediated silver nanoparticle biosynthesis: fungal species, biomolecular drivers, and analytical approaches used for mechanistic characterization.

Study	Fungal Species	Primary Biomolecule Focus	Key Analytical Methods
Yongguang Yin et al., 2016 [39]	*Fusarium oxysporum* CGMCC 3.6787	Superoxide via NADH oxidases	Chemiluminescence, UV-Vis, TEM
Thyerre Santana Da Costa et al., 2025 [36]	*F. oxysporum* VR039 and 07SD	Glyceraldehyde reductase, FAD-oxidoreductase, glucoamylase	Mass spectrometry (Orbitrap), SDS-PAGE
Daniela Ballottin et al., 2016 [34]	*Aspergillus* *tubingensis* AY876924	Glycoamilase, acid phosphatase, serine carboxipeptidase, glucanosyltransferase	LC-MS/MS, Raman, FTIR
Mohammadhassan Gholami-Shabani et al., 2014 [42]	*F. oxysporum* IRAN 31C	Nitrate reductase (214 kDa), NADPH	Ion exchange chromatography, SDS-PAGE, gel filtration
Probin Phanjom & Giasuddin Ahmed, 2017. [19]	*Aspergillus oryzae* MTCC 1846	Nitrate reductase, tryptophan/tyrosine-containing proteins	FTIR, UV-Vis, nitrate reductase assay
Ashraf Sa El Saye et al., 2017 [38]	*A. flavus*	Peroxidase, nitrate reductases	UV-Vis, FTIR, SDS-PAGE, native-PAGE
Sepideh Hamedi et al., 2017 [46]	*Neurospora* *intermedia* PTCC 5291	15 kDa and 23 kDa proteins	UV-Vis, SDS-PAGE, ultrafiltration
Shama Zainab et al., 2023 [49]	*Thermomyces* *lanuginosus* STm	Glucoamylase (67 kDa)	FTIR, LC-MS/MS, SDS-PAGE
Ying-Jie Zeng et al., 2023 [44]	*Fusarium solani* DO7	1,4-alphaglucosidase (72.8 kDa)	Ion exchange chromatography, MALDI-TOF-MS, SDS-PAGE
Nelson Durán et al., 2014 [45]	*Trametes versicolor* CCT 4521	Laccase	UV-Vis, FTIR, Sephacryl/DEAE chromatography
Huda Abdel Hamid & Marwa Obiedallah, 2023. [43]	*A. oryzae* AOry 17	FAD-dependent oxidoreductase, aminopeptidases, amylase, alkaline protease	UV-Vis, TEM, homology modeling, molecular docking
Mohamed Ghareib et al., 2016 [26]	*Cunninghamella* *phaeospora* AUMC 8662	Nitrate reductase (EC 1.6.6.4, 1.06 U/mL), tryptophan	UV-Vis, fluorescence, NR assay
Laryssa Pinheiro Costa Silva et al., 2017 [40]	*Duddingtonia* *flagrans* AC001	Chitinase	FTIR, Raman, Bradford assay, DNS assay
Krishia Rei A. Javier et al., 2023 [30]	*Ganoderma lucidum*	Epicatechin-6- glucoside, ergothioneine, (Z)-9-octadecenamide	UV-Vis, FT-IR, LC-MS/MS
Sepideh Hamedi et al., 2017 [27]	*F. oxysporum* PTCC 5291	Nitrate reductase (259 nmol h^−1^ mL^−1^ at stationary phase)	UV-Vis, Harley assay, Bradford assay
Supriyo Chowdhury et al., 2014 [47]	*Macrophomina* *phaseolina*	85 kDa capping protein, naphthoquinones, anthraquinones	UV-Vis, SDS-PAGE
V. Guleria & J. Saxena, 2023 [52]	*Trichoderma* *harzianum* MTCC 801	Extracellular amylases, nitrate reductases, cysteine thiol groups	UV-Vis, starch hydrolysis assay, TEM
Narasimhamurthy Konappa et al., 2021 [31]	*T. harzianum* MK611661	Flavanones, steroids, alkaloids, phospholipids, harzianopyridone, peptaibols	UV-Vis, FT-IR, LC-MS/MS, HPLC
Liang Ma et al., 2017 [48]	*Penicillium aculeatum* Su1	Proteins (reducing and capping)	FTIR
Abdallah M. Elgorban et al., 2017 [64]	*Curvularia* *pallescens*	Alkaloids, proteins	UV-Vis (general characterization only)
Xingyun Yang & Jian-Yong Wu, 2022 [55]	*Cordyceps sinensis* Cs-HK1	Exopolysaccharides (EPS-1, EPS-2, EPS-3)	HPGPC, HPLC, UV-Vis, DLS, ICP-OES
Maryem Akbal Hasoon et al., 2025 [62]	*Isaria fumosorosea*	Sorbitol, heptadecane, Z-5-nonadecene, heneicosane, and others	GC-MS, UV-Vis, FTIR, FESEM
Ankush Sharma et al., 2022 [63]	*Talaromyces* *purpureogenus* KNUPD2	Terpenoids, phenols, dibutyl phthalate, phthalic acid esters	UV-Vis, FTIR, GC-MS
Abhishek Kaler et al., 2013 [51]	*Saccharomyces* *boulardii*	Proteins/peptides, nitrate reductase	FTIR, UV-Vis, Bradford assay
Pei-Jun Li et al., 2021 [35]	*Aspergillus japonicus* PJ01	Pectinase, CMCase, xylanase, soluble proteins, reducing sugars	FTIR, DNS method, Bradford method
Gemishev et al., 2019 [21]	*Trichoderma reesei* PF	Extracellular Enzymes (unspecified)	FTIR
Wenjie Jian et al., 2017 [54]	*Cordyceps sinensis* Cs-HK1	Galactomannanprotein EPS complex	UV-Vis, HPLC, GPC-MALLS-UV, TEM
Cristina Arrieta Eric et al., 2017 [32]	Moringa oleifera	Griseofulvin, dechlorogriseofulvin, beta-carotenes, flavonoids, tannins	FTIR
Rafael J.V. De Oliveira et al., 2024. [58]	*Aspergillus* *brasiliensis*	Terpenoids, phenols, alkaloids, peptides, polypektides	Phytochemical colorimetric analysis, FTIR
Baharak Mehrdel et al., 2023 [29]	*Agaricus bisporus*	Caffeic acid, quercetin, gallic acid, rosmarinic acid, sinapic acid, syringic acid	HPLC
Al-Zahrani & Saleh Mohammed Al-Garni, 2023 [57]	*Curvularia kusanoi*	Chitosan (exogenous capping agent)	UV-Vis, FTIR, FE-SEM, DLS
Asma Irshad et al., 2024 [56]	*Pleurotus ostreatus*, *P.* *sajor-caju*, *P. sapidus*, *P. columbinus*	Beta-glucan, phenolics	Phenol sulfuric acid test, agar well diffusion
Shama Zainab et al., 2022 [41]	*A. oryzae* SZ1	Not specified	UV-Vis, TEM, XRD

**Table 2:** Effects of physicochemical parameters on fungal-mediated AgNP biosynthesis, including silver precursor concentration, pH, temperature, incubation time, and resulting nanoparticle characteristics.

Study	Species	AgNO_3_	Temp (°C)	Optimal pH	Time	Biomass	Size (nm)
Gemishev et al., 2022 [113]	*T. reesei*	10 mM	40	NR	144 h	10%	2–6
Elmayah et al., 2022 [114]	*T. stipitatus*	7 mmol	25	8	91.2 h	NR	13.95
Naini et al., 2024 [127]	*A. flavus*	4.189 mM	30	NR	8.17 h	0.905 mL CFC	20.5
Al-Hamadani & Kareem, 2018 [88]	*A. niger*	1 mM	30	9	120 h	NR	15–50
Kahraman et al., 2021 [104]	*N. clavispora*	0.25 mM	35	12	72 h	NR	55.15
Elamawi et al., 2018 [109]	*T. longibrachiatum*	1 mM	28	NR	72 h	10 g	10
Kareem et al., 2019 [102]	*A. alternata*	1 mM	40	NR	120 h	NR	7.48–12.15
Youssef et al., 2023 [112]	*P. floridanus*	1 mM	NR	11	NR	30 g/L	11–13
Alamilla-Martínez et al., 2018 [87]	*P. purpurogenum*	2 mM	45	NR	72 h	NR	6.7 (EFS)
El Deeb et al., 2025 [89]	*T. funiculosus*	1 mM	60	5.5	NR	5 g	34.32
Zhu et al., 2024 [91]	*P. polonicum*	80 mM	45	9	48 h	NR	3–25
Narware et al., 2023 [100]	*T. harzianum*	1 mM (1:4)	RT	10	72 h	20 g	43.68
Kumari et al., 2020 [86]	*A. terreus*	1 mM	35	8	24 h	~25 g	~25
Fuinhas Alves & Murray, 2022 [115]	*Six species*	0.5 mM	90	9–12	1 h	NR	3–17
Gemishev et al., 2021 [110]	*T. reesei*	10 mM	29	NR	72 h	20 g	2–6
Wang et al., 2021 [85]	*A. sydowii*	1.5 mM	50	8	NR	20 g	12 ± 2
Abdel-Kareem et al., 2025 [116]	*A. templicola*	1 mM	Elevated	11	10 d	50 g	17.79
Vijayakumar et al., 2024 [90]	*P. adamantinus*	1 mM	37	7	24 h	1 mL extract	50
Selvakumaran et al., 2021 [107]	*A. niger*	1 mM	65	NR	48 h	NR	NR
Trotta et al., 2025 [117]	*P. citrinum, A. niger*	~1.5 mM	25–35	6–8	72 h	75 g/L	31–36
Gupta & Saxena, 2022 [108]	*A. oryzae*	1 mM	28	NR	5 d	10 g	~40
Tomah et al., 2020 [103]	*T. virens*	1 mM	25	7	120 h	10 g	5–50
Fuinhas Alves et al., 2022 [111]	*C. thermophilum*	~0.75 mM	90 (synthesis)	NR	1 h	1 g/10 mL	8.93
Jalal et al., 2018 [101]	*C. glabrata*	1 mM	28	NR	Overnight	NR	2–15

## Data Availability

No new data were created or analyzed in this study. Data sharing is not applicable to this article.

## Abbreviations

The following abbreviations are used in this manuscript:

AgNPs	Silver nanoparticles
AgNO_3_	Silver nitrate
ATR	Attenuated total reflectance
AFM	Atomic force microscopy
CFS	Culture filtrate supernatant
ROS	Reactive oxygen species
DLS	Dynamic light scattering
DRIFTS	Diffuse reflectance infrared Fourier-transform spectroscopy
EDS	Energy-dispersive X-ray spectroscopy
FTIR	Fourier-transform infrared spectroscopy
HRTEM	High-resolution transmission electron microscopy
OFAT	One-factor-at-a-time
PDI	Polydispersity index
RSM	Response surface methodology
SAED	Selected area electron diffraction
SEM	Scanning electron microscopy
SPR	Surface plasmon resonance
TEM	Transmission electron microscopy
UV–Vis	Ultraviolet–visible spectroscopy
XRD	X-ray diffraction
ζ	Zeta potential
ML	Machine learning
